# CDK6 kinase inhibition unmasks metabolic dependencies in BCR::ABL1+ leukemia

**DOI:** 10.1038/s41419-025-07434-1

**Published:** 2025-02-18

**Authors:** Lisa Scheiblecker, Thorsten Klampfl, Eszter Doma, Sofie Nebenfuehr, Omar Torres-Quesada, Sophie Strich, Gerwin Heller, Daniela Werdenich, Waltraud Tschulenk, Markus Zojer, Florian Bellutti, Alessia Schirripa, Sabine Zöchbauer-Müller, Peter Valent, Ingrid Walter, Eduard Stefan, Veronika Sexl, Karoline Kollmann

**Affiliations:** 1 Institute of Pharmacology and Toxicology, University of Veterinary Medicine Vienna, Vienna, Austria; 2 Institute of Medical Biochemistry, Medical University of Innsbruck, Innsbruck, Austria; 3 Tyrolean Cancer Research Institute (TKFI), Innsbruck, Austria; 4 Institute of Molecular Biology and Center for Molecular Biosciences Innsbruck, University of Innsbruck, Innsbruck, Austria; 5 Division of Oncology, Department of Medicine I, Medical University of Vienna, Vienna, Austria; 6 Institute of Morphology, University of Veterinary Medicine Vienna, Vienna, Austria; 7 Division of Hematology and Hemostaseology, Department of Internal Medicine I, Medical University of Vienna, Vienna, Austria; 8 Ludwig Boltzmann Institute for Hematology and Oncology, Medical University of Vienna, Vienna, Austria; 9 University of Innsbruck, Innsbruck, Austria

**Keywords:** Cancer therapy, Cancer metabolism, Acute lymphocytic leukaemia

## Abstract

Metabolic reprogramming and cell cycle deregulation are hallmarks of cancer cells. The cell cycle kinase CDK6 has recently been implicated in a wide range of hematopoietic malignancies. We here investigate the role of CDK6 in the regulation of cellular metabolism in BCR::ABL1+ leukemic cells. Our study, using gene expression data and ChIP-Seq analysis, highlights the contribution of CDK6 kinase activity in the regulation of oxidative phosphorylation. Our findings imply a competition for promoter interaction of CDK6 with the master regulator of mitochondrial respiration, NRF-1. In line, cells lacking kinase active CDK6 display altered mitochondria morphology with a defective electron transport chain. The enhanced cytoplasm/mitochondria ATP ratio paralleled by high pyruvate and lactate levels indicate a metabolic switch to glycolysis. Accordingly, combinatorial treatment of leukemic cells including imatinib resistant cells with the CDK4/6 inhibitor palbociclib and the glycolysis inhibitor 2-deoxyglucose (2-DG) enhanced apoptosis, while blocking cell proliferation in leukemic cells. These data may open a new therapeutic avenue for hematologic malignancies with high CDK6 expression by exploiting metabolic vulnerabilities unmasked by blocking CDK6 kinase activity that might even be able to overcome imatinib resistance.

## Introduction

An altered cell cycle regulation and changes of metabolic pathways are hallmarks of cancer [1]. As cancer cells proliferate in an uncontrolled manner, they need to adjust their metabolism according to their increased need for energy. Otto Warburg was the first to describe the ability of cancer cells to reprogram their metabolism towards glycolysis which is acknowledged as the ‘Warburg effect’ [2]. Normal cells use glycolysis to process glucose to lactate which is further metabolized in the mitochondria. There, electrons get transferred along different respiratory multi-subunit complexes in the inner mitochondrial membrane. During the final oxidative phosphorylation (OXPHOS) step of the electron transport chain (ETC), reduced substrates get oxidized which is coupled to the conversion of ADP into ATP. This mitochondrial respiration is only used under aerobic conditions whereas under oxygen deprived conditions, cells favor glycolysis. Cancer cells can reprogram their metabolism to use mainly glycolysis for energy production even in the presence of oxygen which has been termed “aerobic glycolysis” [2, 3].

Recent evidence points at a role for the cyclin-dependent kinase 6 (CDK6) in cellular metabolism. CDK4/6 kinase inhibition impacts on mitochondrial and glycolytic metabolism in various cancer types [4–6]. Apart from solid cancers, CDK6 has been shown to directly phosphorylate and thereby inhibit key enzymes of glycolysis in acute lymphoid leukemia (ALL) [7]. It remains to be determined how and to which extent CDK6 inhibition alters metabolism of cancer cells and which role CDK6 specifically plays in this context in BCR::ABL1+ leukemia.

CDK6 and its close homologue CDK4 are key players of the G1 phase of the cell cycle. In complex with their regulatory subunits, the D-type cyclins, CDK4/6 phosphorylate the retinoblastoma protein family members (RB) promoting G1 to S-phase progression. Besides its role in cell cycle progression, CDK6 but not CDK4 has been shown to interact with chromatin and to regulate transcription to support tumorigenesis and stem cell functions especially in normal and leukemic hematopoiesis [8–13].

In cancer, deregulation of the individual CDKs is linked to different tumor types with high levels of CDK4 being common in sarcoma, glioma and melanoma [14–16] whereas overexpression of CDK6 is more frequent in hematologic malignancies [17, 18].

B-cell ALL (B-ALL) is among the most common subtypes of ALL and is characterized by uncontrolled proliferation of precursor B-cells. The t(9;22) translocation leading to the *BCR::ABL1* fusion gene is the most frequent cytogenetic abnormality in ALL, with an incidence of 20–30% [19, 20]. Treatment with the specific BCR::ABL1 tyrosine-kinase inhibitor (TKI) imatinib in combination with polychemotherapy has improved therapy responses and survival in these patients. However, some of the ALL patients relapse due to resistance against imatinib and/or other therapies. In these patients, acquired secondary mutations in *BCR::ABL1* that confer resistances against imatinib may be detected [21]. Recently, more potent TKIs against BCR::ABL1 have been developed aiming to overcome imatinib resistance in ALL. However, some of the patients still relapse, especially when ALL cells exhibit BCR::ABL1 T315 or other BCR::ABL1 mutations in compound configuration [22–25]. Therefore, research is seeking novel pathways and targets that may be used to overcome drug resistance in ALL.

It has been shown that survival and proliferation of BCR::ABL1+ cells are dependent on CDK6 expression [21, 26, 27] whereas on the other hand overexpression of CDK6 confers resistance to BCR::ABL1 kinase inhibition [28]. Furthermore, overexpression of CDK6 has been linked to angiogenesis and epigenetic regulation in BCR::ABL1+ ALL [8, 29].

CDK4/6 inhibitors are widely used in the clinics for breast cancer patients and are also in clinical trials for therapy of hematopoietic diseases [30]. However, the clinically used kinase inhibitors show limited therapeutic success, as resistance evolves rapidly. Therefore, second line treatment strategies, like combinatorial treatments, need to be developed.

In this study, we investigate the role of CDK6 in the regulation of mitochondrial metabolism. We found reduced expression of mitochondrial genes and defective mitochondrial functions in BCR::ABL1+ ALL cells harboring a kinase inactive version of CDK6 or lacking CDK6. Furthermore, murine and human leukemic cells require glycolysis and combinatorial inhibition of CDK6 together with the glycolysis regulator 2-deoxy-D-glucose (2-DG) point at a novel therapeutic treatment strategy for BCR::ABL1+ ALL and chronic myeloid leukemia (CML) patients.

## Results

### CDK6 kinase inhibition reduces OXPHOS gene expression

**Fig. 1:**
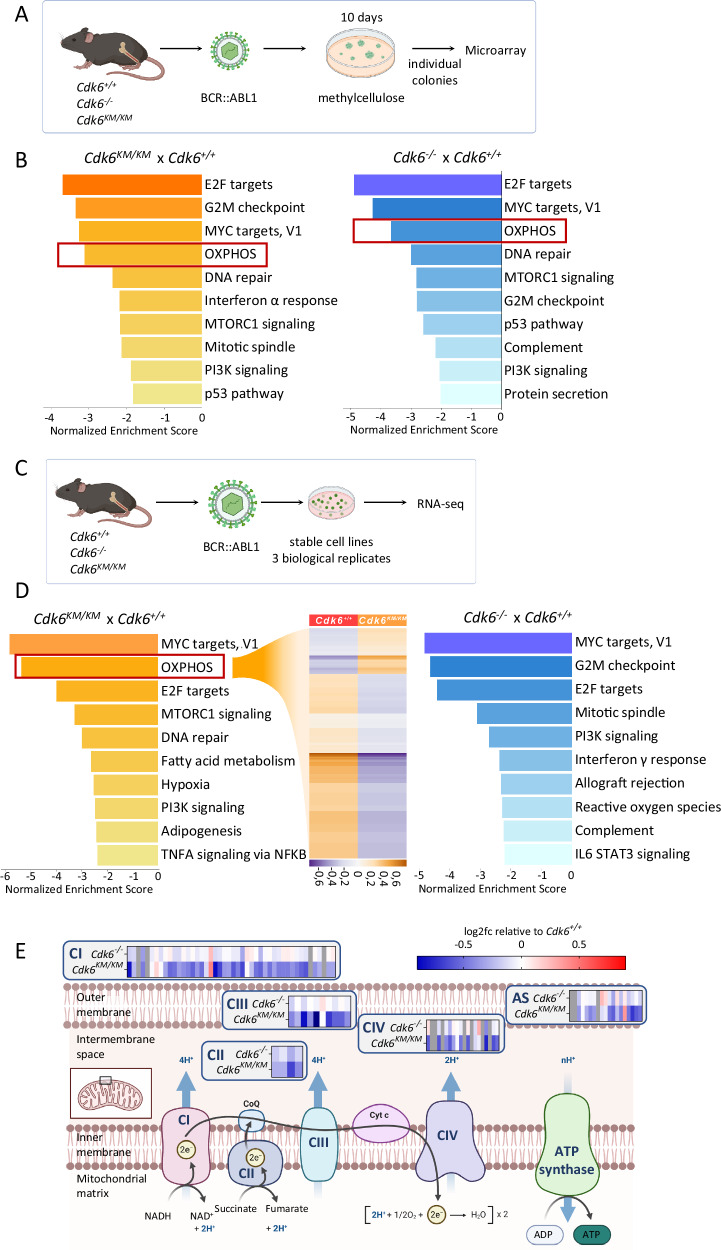
*Cdk6*^*−/−*^ and *Cdk6*^*KM/KM*^ cells have OXPHOS genes down regulated. **A** Schematic workflow of the generation of BCR::ABL1 transformed colonies followed by microarray analysis. **B** Normalized enrichment scores of HALLMARK gene sets in *Cdk6*^*KM/KM*^ versus *Cdk6*^*+/+*^ (left) and *Cdk6*^*−/−*^ versus *Cdk6*^*+/+*^ (right); *n* = 4 biological replicates per genotype. **C** Schematic workflow of stable BCR::ABL1+ cell line establishment followed by RNA-seq analysis. **D** Results from a gene set enrichment analysis. Bar charts depict normalized enrichment scores of HALLMARK gene sets, *Cdk6*^*KM/KM*^ versus *Cdk6*^*+/+*^(left) and *Cdk6*^*−/−*^ versus *Cdk6*^*+/+*^ (right). The heatmap shows mean normalized expression (read counts) of genes from the OXPHOS gene set in *Cdk6*^*KM/KM*^ versus *Cdk6*^*+/+*^ cells; *n* = 3 cell lines per genotype. **E** Heatmaps depicting log2 fold changes of RNA-seq data from *Cdk6*^*KM/KM*^ and *Cdk6*^*−/−*^ relative to *Cdk6*^*+/+*^ of transcripts encoding the different subunits of the ETC complexes (CI – CIV); AS = ATP synthase. The graphic has been adapted from “Electron Transport Chain”, by BioRender.com (2022). Retrieved from https://app.biorender.com/biorender-templates.

High levels of CDK6 are critical for proliferation and survival of BCR::ABL1+ ALL cells [21, 26, 27, 31]. Analysis of the publicly available Microarray Innovations in Leukemia (MILE) study (GSE13159) further validated the upregulation of CDK6 in patients with BCR::ABL1+ ALL (Supplementary Fig. S1A) [32, 33]. CDK6 regulates transcription in a kinase-dependent and -independent manner [8–13]. To understand the consequences of CDK6-mediated gene expression, we made use of our transcriptomics data obtained in BCR::ABL1 transduced CDK6 wildtype (*Cdk6*^*+/+*^), kinase inactive Cdk6K43M (*Cdk6*^*KM/KM*^), and knockout (*Cdk6*^*−/−*^) primary bone marrow cells (Fig. 1A) [12, 34, 35], resembling an early stage of BCR::ABL1+ ALL. *Cdk6*^*KM/KM*^ cells harbor a kinase inactive mutant of CDK6 and at least partially mimic treatment with the clinically used CDK4/6 kinase inhibitor, palbociclib. This dataset was obtained during an early phase of transformation allowing us to study the onset of the disease. The canonical function of CDK6 was validated by the top down-regulated cell cycle related pathways in *Cdk6*^*KM/KM*^ as well as in *Cdk6*^*−/−*^ cells (Fig. 1B). Besides that, OXPHOS was one of the most significantly down-regulated pathways in both, *Cdk6*^*KM/KM*^ and *Cdk6*^*−/−*^ freshly transduced precursor cells (Fig. 1B, Supplementary Fig. S1B).

To examine later progression stages of the disease, we revisited transcriptomic data from stably transformed BCR::ABL1+ cell lines with and without CDK6 deletion generated by the CRISPR-Cas9 system (Supplementary Fig. S1C) [29]. This model allows the presence of CDK6 during transformation and CDK6 deletion was performed upon establishment of stable cell lines. This setting is thought to better resemble the situation in patients at the beginning of therapy as their disease is usually already advanced at this time. Pathway analysis of differentially expressed genes confirmed the OXPHOS gene set among the most significantly down-regulated pathways upon CDK6 deletion (Supplementary Fig. S1D).

As a third approach, transcriptomic analysis of stable BCR::ABL1+ cell lines expressing *Cdk6K43M* (Fig. 1C) underlined the OXPHOS pathway being under the top down-regulated ones compared to CDK6 wildtype cell lines (Fig. 1D, left panel). Of note, the OXPHOS system was not identified among the top deregulated pathways in stable CDK6 knockout cell lines, which may indicate a potential compensatory mechanism when CDK6 is lost long-term (Fig. 1D, right panel). In depth analysis of RNA-seq data (Fig. 1C) showed that transcripts encoding subunits of all complexes of the ETC were affected by kinase inactive CDK6 and partially by the loss of CDK6 (Fig. 1E).

### CDK6 binds to promoters of OXPHOS genes

**Fig. 2:**
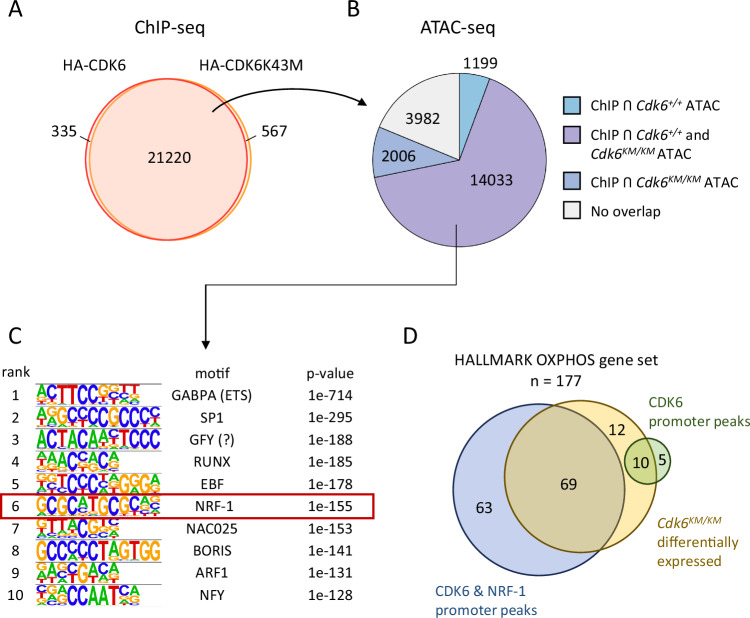
CDK6 binds to promoters of OXPHOS genes. **A** Venn diagram showing the overlap of CDK6-ChIP sequencing peaks of BCR::ABL1+ HA-Cdk6 and HA-Cdk6K43M cell lines; *n* = 2 per condition. **B** Venn diagram showing the overlap of ChIP-seq peaks and open chromatin regions from ATAC-seq of wildtype CDK6 and kinase inactive CDK6; *n* = 2 per genotype. **C** Top 10 hits from a transcription factor motif analysis of overlapping chromatin regions from ChIP-seq and ATAC-seq. **D** Venn diagram showing the overlap of genes from the HALLMARK OXPHOS gene set (*n* = 177) and differentially expressed genes from RNA-seq, genes harboring a CDK6 ChIP-seq or/and an NRF-1 ChIP-seq peak.

CDK6 has been shown to bind chromatin-associated complexes and thereby regulate gene transcription besides lacking a DNA-binding domain [8–12]. We hypothesized that the downregulation of OXPHOS gene expression upon loss of CDK6 kinase function is a consequence of altered transcriptional regulation by *Cdk6*^*KM/KM*^. ChIP-seq experiments in BCR::ABL1+ cell lines were performed to test this concept using cells expressing either wildtype or kinase inactive CDK6. This analysis allows to determine if kinase activity of CDK6 alters chromatin binding. 98% of all sites occupied by wildtype CDK6 were also bound by kinase inactive CDK6, showing that CDK6 interaction with the DNA does not require kinase function (Fig. 2A). Overlapping the CDK6-ChIP-seq results with ATAC-seq allows to prioritize peaks in chromatin regions that are accessible and potentially transcriptionally active. We identified 14 033 regions bound by CDK6 and open as defined by ATAC-seq in *Cdk6*^*+/+*^ and *Cdk6*^*KM/KM*^ cells (Fig. 2B). More than 50% of these open regions are located within promoters (Supplementary Fig. S2A). Transcription factor (TF) motif enrichment analysis revealed ETS, SP1 and NFY motifs (Fig. 2C) among the top enriched motifs. All of these TFs have recently been described as co-regulators of CDK6-mediated transcription [12, 29]. In addition, we found the NRF-1 motif among the top 10 enriched motifs. NRF-1 is a master regulator of nuclear genes required for the function of mitochondrial respiration [36, 37]. We confirmed CDK6 binding at NRF-1 target sites using a publicly available NRF-1 ChIP-seq dataset of transformed B-cells [38]. In total, around 50% of CDK6 binding sites were also bound by NRF-1 (Supplementary Fig. S2B). To analyze if the differentially expressed OXPHOS genes between *Cdk6*^*+/+*^ and *Cdk6*^*KM/KM*^ cells from our RNA-seq (Fig. 1D) harbor a CDK6 peak as well as an NRF-1 peak in the same position of their promoter region, we overlapped the three data sets. Roughly 40% of genes (69 from 177) defining the OXPHOS gene set were differentially expressed in *Cdk6*^*KM/KM*^ cells and showed overlapping CDK6 and NRF-1 peaks at their promoter regions (Fig. 2D). This finding prompted us to ask whether CDK6 directly interacts with NRF-1 to regulate transcription. As we failed to obtain any evidence for a direct interaction between CDK6 and NRF-1 by co-immunoprecipitation experiments (Supplementary Fig. S2C) we speculate that CDK6 and NRF-1 antagonistically interact with OXPHOS gene promoters and that kinase inactive CDK6 has a repressive function.

### Kinase inactive CDK6 alters cell respiration

**Fig. 3:**
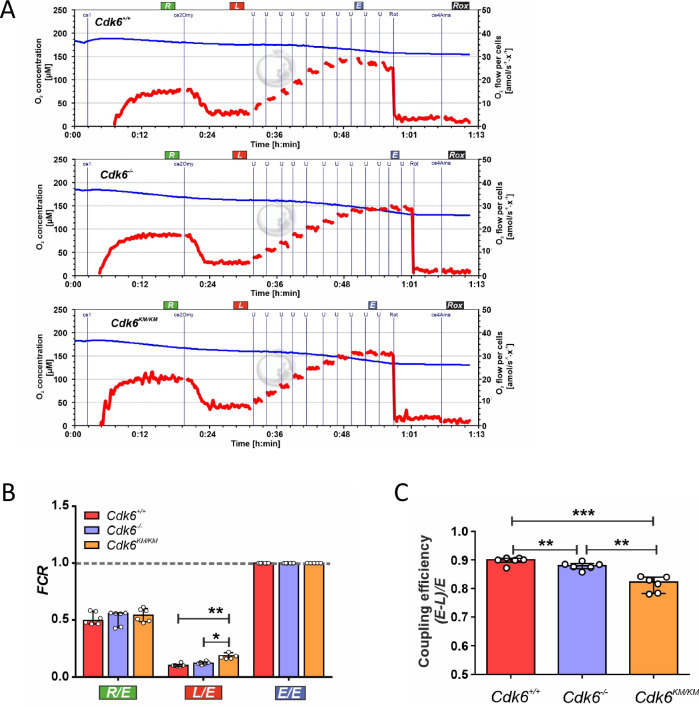
*Cdk6*^*KM/KM*^ cells show mitochondrial dysfunction due to dyscoupled respiration. **A** Representative O2k traces showing O_2_ concentration (left axis) and O_2_ flow per cells (right axis) from BCR::ABL1 transformed living cells analyzed using high-resolution respirometry. **B** Evaluation of the flux control ratio *FCR* corrected by *Rox* (residual oxygen consumption). **C** Evaluation of the biochemical coupling efficiency *(E-L)/E*. Coupling states: ROUTINE (*R*), LEAK respiration (*L*) and ET capacity (*E*). Values are represented as median ±IQR (50% range). *n* = 6; *p ≤ 0.05, **p ≤ 0.01, ***p ≤ 0.001.

To investigate the effects of CDK6 kinase activity on mitochondrial function, we applied high-resolution respirometry (HRR) on the *Cdk6*^*+/+*^, *Cdk6*^*−/−*^ and *Cdk6*^*KM/KM*^ cells. We evaluated O_2_ consumption (flows), flux control ratio (*FCR*) and coupling efficiencies *(E-L)/E* to determine the mitochondrial coupling control of electron flow to ATP production. *FCR* gives a fingerprint of mitochondrial coupling control independent of changes in mitochondrial content, cell volume and sample amount [39–41]. The analysis of the O_2_ flows revealed that the *Cdk6*^*KM/KM*^ cells have an increased respiration in all coupling control states: the physiological respiration ROUTINE (*R*), the non-phosphorylating resting or intrinsic uncoupled LEAK respiration (*L*) and the maximum experimentally induced respiration or ET capacity (*E*) when compared to *Cdk6*^*+/+*^ cells (Fig. 3A, Supplementary Fig. S3A). The leak state is associated with uncoupled or dyscoupled respiration when electron flow is not coupled to ATP production but is still used to pump protons to compensate for the proton leak [42]. The proton leak is associated with an uncoupled or dyscoupled respiration and reflected how the electron flow is coupled to ATP synthesis. An enhanced LEAK respiration might reflect higher intrinsic uncoupling or, under pathological/ toxicological conditions, a dyscoupling, which is associated with a mitochondrial dysfunction [39, 41]. In analyses of the *FCR* we detected a significant increase of the *L/E* ratio in *Cdk6*^*−/−*^ and *Cdk6*^*KM/KM*^ cells (Fig. 3B) which, together with the increase in the O_2_ flows in LEAK respiration (Supplementary Fig. S3A) are indicative for a mitochondrial dyscoupling. These results are supported by the analysis of the coupling efficiency (Fig. 3C) which displayed a significant decrease in the *Cdk6*^*−/−*^ and *Cdk6*^*KM/KM*^ cells when compared to *Cdk6*^*+/+*^ cells. In all the comparisons the *Cdk6*^*KM/KM*^ mutant exhibited the strongest effect.

Our results unveiled that cells expressing kinase inactive CDK6 display mitochondrial failures which are compensated by an increase of cell respiration.

### Cells with kinase inactive CDK6 show altered mitochondria morphology

**Fig. 4:**
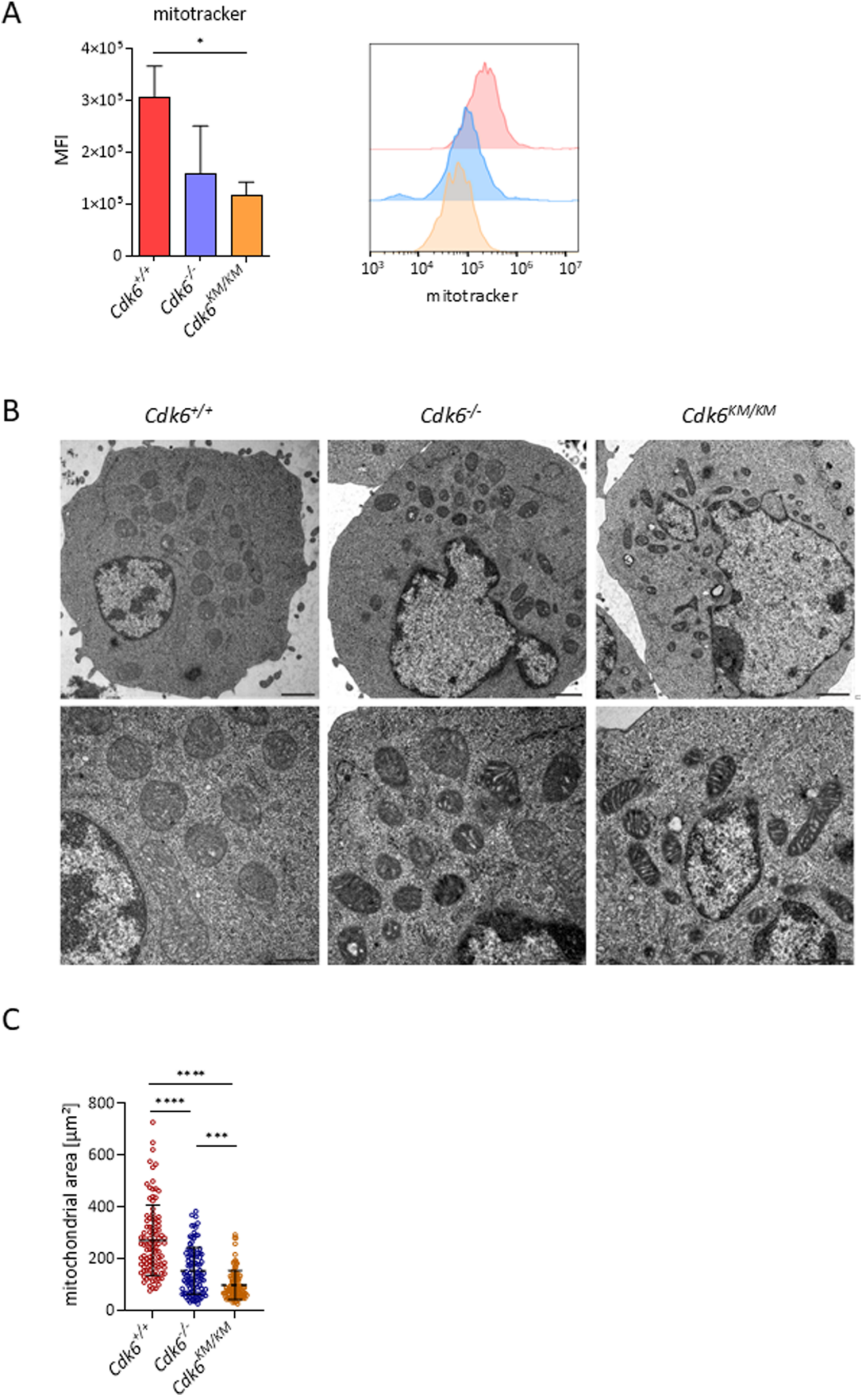
*Cdk6*^*KM/KM*^ cells have smaller and condensed mitochondria. **A** Mean fluorescence intensity (MFI) of mitotracker stainings of BCR::ABL1+ cell lines depicted as bar graph (left) and histogram overlay (right); *n* = 3 per genotype. Data are depicted as mean ± SD; *p ≤ 0.05. **B** Representative transmission electron microscope pictures of ultra-thin cuts of BCR::ABL1+ cell lines (scale bar 1000 nm (upper panel) and 500 nm (lower panel)). **C** Quantification of the mitochondrial area of transmission electron microscopy pictures of ultra-thin cuts (*n* = 100 mitochondria per genotype). Data are depicted as mean ± SD; ***p ≤ 0.001; ****p ≤ 0.0001.

One way to examine mitochondrial function is by performing mitotracker experiments. The mitotracker dye passively diffuses across the plasma membrane and accumulates in active mitochondria. The accumulation depends on the membrane potential and is used as an indicator of mitochondrial mass. Reduced mitotracker signals were detected in *Cdk6*^*−/−*^ cells and a significant reduction was observed in *Cdk6*^*KM/KM*^ cells (Fig. 4A). To analyze a possible correlation of proliferation and OXPHOS regulation, we examined the proliferation rate and mitotracker levels of BCR::ABL1+ cell lines with and without CDK4 deletion generated by the CRISPR-Cas9 system. Cells with the CDK4 deletion showed a significantly reduced proliferation rate compared to its controls, however mitotracker signal did not change (Supplementary Fig. S4A, B). Deleting CDK6’s close homolog CDK4 demonstrated that the observed dysregulation in OXPHOS is specific to CDK6, and that it is not merely a consequence of reduced proliferation.

Transmission electron microscopy showed morphologically changed, condensed mitochondria with significantly smaller sizes in *Cdk6*^*KM/KM*^ and mitochondria of mixed phenotypes (normal and condensed) and sizes in *Cdk6*^*−/−*^ cells (Fig. 4B, C). Interestingly, in solid cancers short-term CDK4/6 inhibition enhanced mitochondrial mass and mitochondrial numbers [4, 6]. Our data in leukemia suggest that long-term inhibition of CDK6 alone might reverse this phenotype, highlighting the need for further studies on long-term CDK6 inhibition in solid cancers.

### Cells with kinase inactive CDK6 have an increased need for aerobic glycolysis

**Fig. 5:**
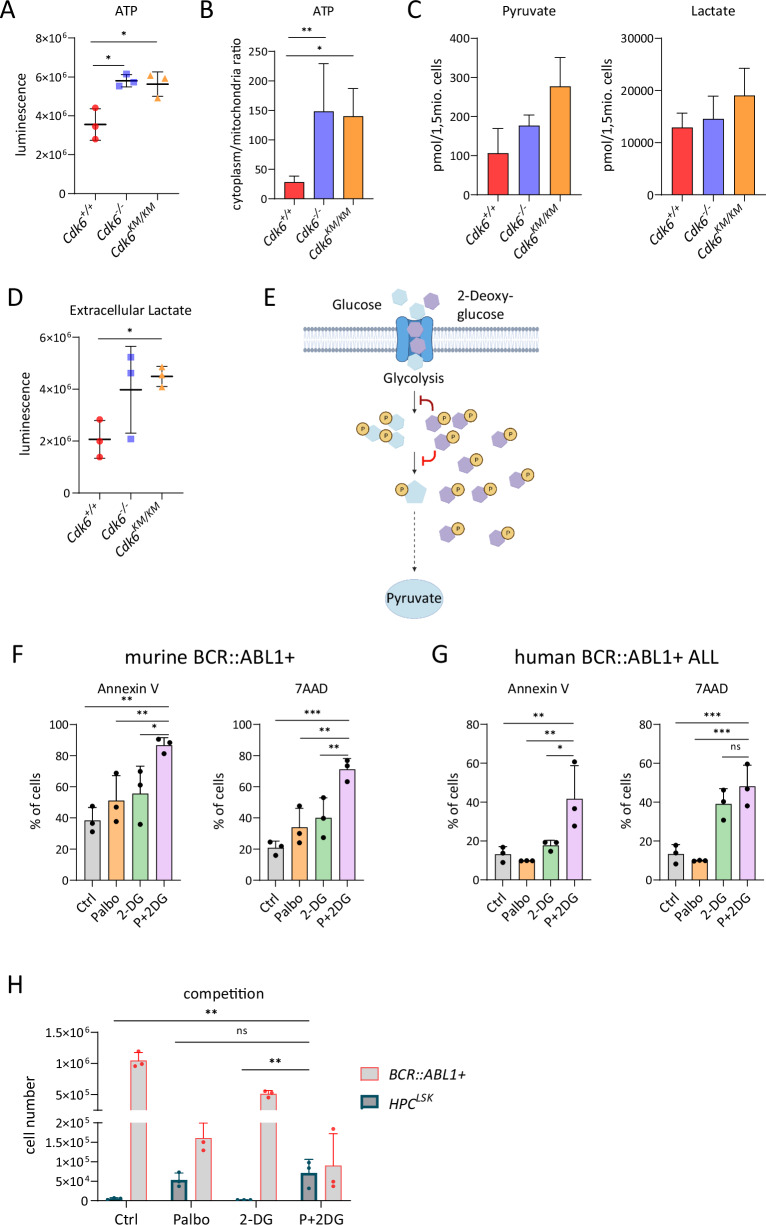
*Cdk6*^*KM/KM*^ cells have an increased need for aerobic glycolysis. **A** ATP levels determined by CellTiter-Glo® Luminescent Cell Viability Assay; *n* = 3 cell lines per genotype. Data are depicted as mean ± SD; *p ≤ 0.05. **B** Cytoplasm/mitochondria ratio of ATP levels as determined by CellTiter-Glo® Luminescent Cell Viability Assay after separation of mitochondria and cytoplasmatic fractions; *n* = 3 cell lines per genotype in duplicates. Data are depicted as mean ± SD; **p ≤ 0.01. **C** Pyruvate (left) and lactate (right) levels as determined by high-throughput metabolite analysis in BCR::ABL1+ cell lines; *n* = 3 cell lines per genotype. Data are depicted as mean ± SD; *p > 0.05. **D** Extracellular lactate levels measured with Lactate-Glo™ Assay; *n* = 3 cell lines per genotype; Data are depicted as mean ± SD; *p ≤ 0.05. **E** Scheme of the inhibitory mechanism of 2-DG. 2-DG and glucose get competitively transported into the cell where they get both phosphorylated in the first step of glycolysis. However, 2-DG-6-P cannot be further metabolized, accumulates within the cell and inhibits both up- and downstream enzymes of glycolysis. **F** AnnexinV/7AAD stainings of BCR::ABL1+ cells on day 13 of a proliferation experiment (see Supplementary Fig. S5E); *n* = 3 cell lines per genotype. Data are depicted as mean ± SD; *p ≤ 0.05, **p ≤ 0.01, ***p ≤ 0.001. **G** AnnexinV/7AAD stainings of human BCR::ABL1+ SUPB15 cells on day 14 of proliferation experiment (see Supplementary Fig. S5H); *n* = 3 replicates. Data are depicted as mean ± SD; *p ≤ 0.05, **p ≤ 0.01, ***p ≤ 0.001. **H** Number of living HPC^LSK^ cells compared to murine BCR::ABL1+ cells in a co-culture setting with the indicated treatment at day 10; *n* = 3 replicates. Data are depicted as mean ± SD. HPC^LSK^ fraction relative to BCR::ABL1+ was compared between treatment conditions; **p ≤ 0.01, ns not significant.

Mitochondrial dysfunctions are frequently associated with changes in intracellular ATP levels. In contrast to our expectation, ATP levels were significantly increased in *Cdk6*^*−/−*^ and *Cdk6*^*KM/KM*^ cells (Fig. 5A). This finding prompted further investigations by separating mitochondria from the cytoplasm to analyze ATP levels in both compartments. The cytoplasm/mitochondria ratio of ATP showed significantly higher ATP levels in the cytoplasm of *Cdk6*^*−/−*^ and *Cdk6*^*KM/KM*^ cells compared to *Cdk6*^*+/+*^ cells (Fig. 5B). Mitochondria and cytoplasmic ATP levels normalized to the total protein content of their respective fraction showed that mutant cells have less mitochondrial and increased cytoplasmic levels of ATP. These data support the hypothesis of a metabolic switch when CDK6 is inhibited (Fig. S5A).

The concept of cancer cells switching their metabolism to increased aerobic glycolysis instead of relying on OXPHOS for ATP generation is widely acknowledged as the ‘Warburg effect’ [2] (Supplementary Fig. S5B). The reduced functionality of *Cdk6*^*−/−*^ and *Cdk6*^*KM/KM*^ mitochondria and the high levels of cytoplasmic ATP led us to conclude that CDK6 inhibition enforces and enhances the metabolic switch towards the Warburg effect. In line with our concept, we detected elevated levels of pyruvic acid and lactic acid in *Cdk6*^*KM/KM*^ cells and to a lesser extent in *Cdk6*^*−/−*^ cells, using high-throughput metabolite analysis but the differences did not reach statistical significance (Fig. 5C). These observations were confirmed by measuring extracellular lactate levels, which were also significantly elevated in *Cdk6*^*KM/KM*^ cells (Fig. 5D). Even though not significant, a trend mimicking the increased lactate production of CDK6 mutant cells could be seen upon treatment of *Cdk6*^*+/+*^ cells with the CDK4/6 kinase inhibitor palbociclib (Supplementary Fig. S5C).

A recent publication indicated an inverse correlation between *CDK6* and the prominent glucose transporter GLUT3*/SLC2A3* expression levels in acute lymphoid leukemia patient samples [27]. In validation, *Slc2a3* was higher expressed in cells harboring the kinase inactive CDK6 variant (Supplementary Fig. S5D). *Cdk6*^*−/−*^ cells showed a weaker effect.

To investigate if the CDK6-dependent metabolic/glycolytic regulation may be therapeutically exploited we tested combinatorial treatments with palbociclib and the glucose analog 2-DG. 2-DG blocks glycolysis and ATP generation (Fig. 5E) and is currently in clinical trials as combinatory agent for solid cancers [43]. Murine BCR::ABL1+ *Cdk6*^*+/+*^ cells were treated with palbociclib or 2-DG alone or in combination and cell numbers were assessed over time (Supplementary Fig. S5E). Cells treated with the combination of palbociclib and 2-DG showed a reduced growth rate compared to single agent treated or untreated control cells.

Differences became prominent after one week, which supports the concept that long-term inhibition of CDK6 is required to unmask the metabolic vulnerability to glycolysis inhibition. Analysis at day 13 uncovered a significantly higher G1 arrest of the cell cycle upon double treatment (Supplementary Fig. S5F) and higher numbers of Annexin V and 7AAD positive cells indicative for early apoptotic and dead cells, respectively (Fig. 5F, Supplementary Fig. S5G). To test the relevance of our finding in human cellular systems, we repeated the combinatorial treatment with palbociclib and 2-DG in the BCR::ABL1+ ALL cell line SUPB15. These experiments confirmed the reduced proliferation rate (Supplementary Fig. S5H) and enhanced apoptosis upon combinatorial treatment (Fig. 5G, Supplementary Fig. S5I).

To test the impact of the drug treatments on untransformed cells, we took advantage of the murine hematopoietic progenitor cell line (HPC^LSK^) in a co-culture system with the murine BCR::ABL1+ cells to create a bone marrow niche-like model [44] (Supplementary Fig. S5J). In this competitive setting, HPC^LSK^ cells have the highest living cell number and best outgrowth with the combinatorial treatment compared to the control and single treatments (Fig. 5H, Supplementary Fig. S5K). These experiments confirmed that BCR::ABL1+ cells had reduced growth rate and survival following the combinatorial treatment, highlighting a higher toxicity for transformed than untransformed cells.

### Combined inhibition of CDK6 and glycolysis increases apoptosis in CML

**Fig. 6:**
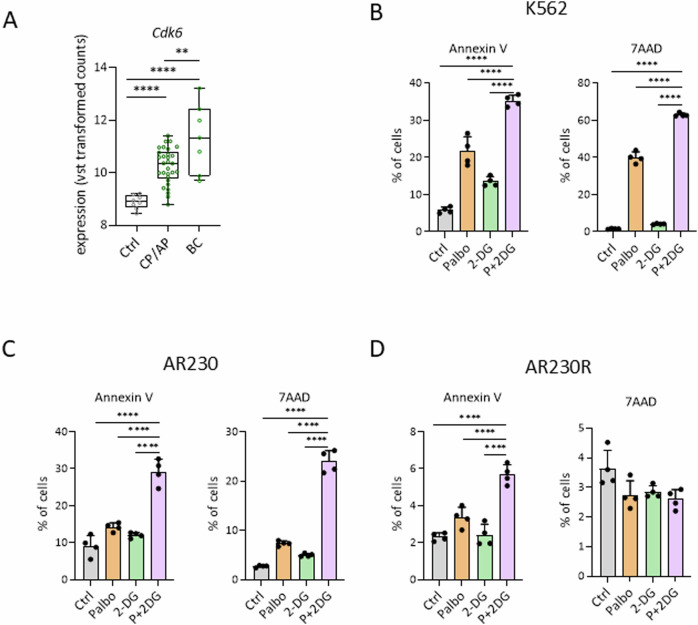
Combined inhibition of CDK6 and glycolysis increases apoptosis in CML. **A** *Cdk6* expression (variance of standard transformed expression values) in patient samples with different stages of CML and healthy control samples. Control (Ctrl) *n* = 8, chronic/accelerated phase (CP/AP) *n* = 30, blast crisis (BC) *n* = 7. **p ≤ 0.01, ****p ≤ 0.0001. AnnexinV/7AAD stainings of human K562 (**B**) and AR230 (**C**) CML cells on day 13 and AR230R (**D**) on day 11 of proliferation experiments (see Supplementary Fig. S6A, B, E); *n* = 4 replicates. Data are depicted as mean ± SD; ****p ≤ 0.0001.

The *BCR::ABL1* fusion oncogene is not only common in ALL but represents a hallmark feature in chronic myeloid leukemia (CML). To evaluate the role of CDK6 in this disease context, we analyzed RNA-Seq data of a previously published CML patient cohort, including CML patients in chronic phase (CP-CML), accelerated phase (AP-CML) and blast crisis (BC-CML) as well as in controls [45]. Strikingly, CDK6 expression correlated with disease progression (Fig. 6A). To test the efficacy of the combined palbociclib and 2-DG treatment in CML, we used the human BCR::ABL1+ cell lines K562 and AR230. In line with the ALL cell lines, the CML cells showed a reduced proliferation rate (Supplementary Fig S6A and S6B) and enhanced apoptosis upon combinatorial treatment (Fig. 6B, C, Supplementary Fig. S6C, D).

This also held true for imatinib resistant AR230 cell lines [46]. As acquired resistance to imatinib and related compounds frequently provokes relapse in patients the increased apoptosis and reduced proliferation upon combinatorial treatment is of high clinical relevance (Fig. 6D, Supplementary Fig. S6E, F).

In summary, our data in B-ALL cells reveal a reduced mitochondrial function, enhanced levels of glycolysis paralleled by higher expression of glucose importers in CDK6 deficient and CDK6 kinase inactive cells. These findings are indicative for a CDK6 kinase-dependent switch in metabolic dependencies which we proved to be relevant for apoptosis induction and which might even be expanded to the context of CML.

## Discussion

Targeting CDK6 in cancer represents an attractive therapeutic option as CDK6 activity is frequently enhanced in disease and CDK4/6 kinase inhibitors are available for clinical use. Inhibiting CDK6 kinase function alone is not sufficient and requires combinatorial treatments. We here describe a novel option for combination and show that CDK6 balances metabolism and that its kinase inhibition leads to a switch to glycolysis in lymphoid leukemia.

Our results identified the OXPHOS gene set as one of the top down-regulated pathways in BCR::ABL1+ cells lacking CDK6 kinase activity. Lower expression of ETC components, at least partially, impacts on the function of mitochondria as shown in HRR experiments. Leukemic cells show a more condensed mitochondrial phenotype upon CDK6 kinase inhibition, which contrasts previous studies in solid cancer. Short-term CDK4/6 kinase inhibition enhanced mitochondrial mass and mitochondrial numbers in those cases [4, 6]. We speculate that the observed mitochondrial defects in BCR::ABL1+ cells are long-term results which are not mimicked by short-term CDK6 kinase inhibition. We hypothesize that CDK2 activity is upregulated upon persistent CDK6 kinase inhibition, to allow proliferation as frequently observed in palbociclib resistant patients [47]. This potential interplay of the two kinases may provoke the downregulation of OXPHOS genes as they both interfere with transcription of mitochondrial genes. We detected enhanced chromatin binding of CDK6 to target sites of the transcriptional regulator of mitochondrial genes, NRF-1. A previous study showed that phosphorylation of S136 within the DNA binding domain of NRF-1 releases NRF-1 from the DNA and thereby reduces the expression of target genes. The critical phosphorylation site was identified as a target of CDK2 [48].

While it seems that mitochondrial activity is influenced by long-term CDK6 inhibition, enhanced glycolysis is already observed upon short-term palbociclib treatment [4–6]. We found pyruvic acid and lactic acid enriched in *Cdk6*^*−/−*^ and *Cdk6*^*KM/KM*^ in line with studies in solid cancers [4–6]. In T-ALL, it was shown that CDK6 in complex with cyclin D3 inhibits glycolysis by directly phosphorylating its key enzymes which is in line with the enhanced glycolytic rate observed in *Cdk6*^*KM/KM*^ cells in this study [7].

CDK6 itself lacks a DNA binding motif and requires co-factors to bind DNA such as NRF-1. We failed to detect any interaction between the transcription factor NRF-1 and CDK6 despite binding the same promoter regions of NRF-1 target genes. We thus propose a model of competing function between these two factors.

This model implies that CDK6 interacts with other transcription regulators to balance mitochondrial functionality. One known co-factor of CDK6-mediated gene expression is STAT3. A STAT3-CDK6 complex induces the expression of the CDK6 inhibitor *p16*^*INK4a*^ [8]. STAT3 has also been reported to impact mitochondrial transcription as well as respiration [49, 50]. Even though its localization in the mitochondria and direct binding to mitochondrial DNA is debated [51], it could influence mitochondrial function via CDK6-mediated transcriptional programs. However, if CDK6 and STAT3 cooperate to regulate oxidative respiration remains to be determined in more detail.

Resistance development is one of the major limitations of targeted therapy. In patients treated with imatinib or other TKIs, secondary mutations in *BCR::ABL1* conferring resistance can be found [21]. Especially mutations interfering with the compound configuration of TKIs, like BCR::ABL1 T315 are difficult to target. However, CML clones expressing BCR::ABL1 T315 show decreased proliferation upon palbociclib treatment underlining the role of CDK6 in this disease context and suggesting that suppression of CDK4/CDK6 may be a promising concept to overcome BCR::ABL1 T315I-associated TKI resistance [52].

Finally, we suggest a therapeutic strategy of combinatorial treatment with CDK4/6 kinase inhibitors and glycolysis inhibitors, like 2-DG. Our data suggest that BCR::ABL1+ ALL and CML cells respond to this treatment strategy with increased apoptosis and reduced proliferation. This therapeutic regimen might also bring an advantage for imatinib resistant leukemias.

## Materials and methods

### Electron microscopy

Cells were pelleted and fixed in 3% glutaraldehyde. Pellets were pre-embedded in HistoGel (Epredia), postfixed for 2 h in 1% osmium tetroxide (Electron Microscopy Sciences, Hatfield, PA, USA) followed by dehydration and embedded in epoxy resin (Serva, Mannheim, Germany). Ultra-thin sections (70 nm) were cut and mounted on copper grids and contrasted with uranyl acetate (Fluka Chemie GmbH, Buchs, Switzerland) and lead citrate (Merck, Darmstadt, Germany). For imaging, a transmission electron microscope EM 900 (Zeiss) equipped with a slow-scan CCD 2 K wide-angle dual speed camera (TRS, Moorenweis, Germany) and Image Sys Pro software (TRS) were used. Mitochondrial area of 100 mitochondria per genotype was measured on representative electron micrographs using the ImageJ software (Fiji). Ordinary one-way ANOVA test with multiple comparisons was performed to evaluate statistical significance between *Cdk6*^+/+^, *Cdk6*^−/−^ and *Cdk6*^*KM/KM*^.

### Proliferation curves

Cells were seeded and proliferation was monitored either in an untreated state or cells were treated with either one agent or a combination of both with the following concentrations every 48 h. BCR::ABL1+ transformed murine cell lines: 500 nM palbociclib, 250 µM 2-DG; SUPB15: 250 nM palbociclib, 100 µM 2-DG; K562, AR230: 750 nM palbociclib, 1 mM 2-DG; AR230R: 750 nM palbociclib, 100 µM 2-DG. Cells were split every 3-4 days according to their density. Cell numbers were determined in regular intervals with the CytoFLEX S (Beckman Coulter, Fullerton, CA, USA). Data were analyzed using CytExpert and Graphpad Prism. Paired, two-tailed t-test was performed to evaluate statistical significance between *Cdk4*^*+/+*^ and *Cdk4*^*−/−*^.

### Flow cytometry

Cell cycle was analyzed via propidium iodide staining. Apoptosis was measured using the eBioscience Annexin V Apoptosis Detection Kit eFluor 450, 7-AAD (Invitrogen, Carlsbad, CA, USA) according to the manufacturer’s instructions. For flow cytometry, CytoFLEX S (Beckman Coulter) was used. Data were analyzed using the CytExpert software and Graphpad Prism. Statistical significance was determined using linear models in R, where we modeled % positive cells as a function of treatment.

### ChIP sequencing and data analysis

In BCR::ABL1+ *Cdk6*^*−/−*^ cell lines, an HA-tagged version of Cdk6 or Cdk6K43M was overexpressed. CDK6 chromatin immunoprecipitation (ChIP) was performed using an antibody against HA (ab9110, abcam, Cambridge, UK) as described previously [12]. Intracellular proteins were crosslinked with DSG (20 min, room temperature) and protein-DNA interactions were fixed with 1% formaldehyde (10 min, room temperature). Termination of the crosslinking procedure was performed using glycine. For immunoprecipitation, 70 μl Dynabeads Protein G magnetic beads (Invitrogen) were used. A sequencing library of immunoprecipitated DNA was generated and sequencing was performed using the Illumina HiSeq3000/4000 platform. ChIP-seq data reported in this article are deposited in the GEO database (accession: GSE264219). Raw sequencing reads were quality controlled using the FASTQC software (version 0.11.5). Trimmomatic (version 0.36) was used to perform adapter trimming and quality-based read filtering. Next, the filtered reads were mapped against the mm10 mouse reference genome (Gencode M13) using bwa-mem (v0.7.15) and blacklisted regions were removed with bedtools subtract (v2.26.0). Multimappers as well as reads with low mapping quality were removed using samtools (v1.3.1). Peak calling was performed with MACS2 (v2.1.0) using default parameters. The R package Diffbind (version 2.15.1) was used to define genomic regions for analysis of peak overlaps between HA-Cdk6 and HA-Cdk6K43M samples. We defined genomic regions, in which both HA-Cdk6 samples had peaks and none of the two HA-Cdk6K43M samples had peaks as uniquely bound by HA-Cdk6. Likewise, we defined genomic regions uniquely bound by HA-Cdk6K43M. Genomic regions in which at least three of the four samples had peaks were regarded as bound by both Cdk6 variants.

Other methods are described in detail in the Supplementary Materials and Methods.

## Supplementary information

supplemental material

## Data Availability

The accession numbers for the data sets reported in this paper are: GSE87420, GSE145220, GSE156966, GSE266394, GSE266393, GSE264219, GSE267912, GSE13159.
