# Supplementary material for: CDK6 kinase inhibition unmasks metabolic dependencies in BCR::ABL1+ leukemia
Source: Cell Death Dis. 2025 Feb 18;16(1):107. doi: 10.1038/s41419-025-07434-1 (PMC11836434; doi:10.1038/s41419-025-07434-1)

Data Supplement

CDK6 kinase inhibition unmasks metabolic dependencies in BCR::ABL1+ leukemia

Lisa Scheiblecker, Thorsten Klampfl, Eszter Doma, Sofie Nebenfuehr, Omar Torres-Quesada, Sophie Strich, Gerwin Heller, Daniela Werdenich, Waltraud Tschulenk, Markus Zojer, Florian Bellutti, Alessia Schirripa, Sabine Zöchbauer-Müller, Peter Valent, Ingrid Walter, Eduard Stefan, Veronika Sexl and Karoline Kollmann

Supplementary Figure Legends

Figure S1. *Cdk6$^{-/-}$* and *Cdk6$^{KM/KM}$* cells have OXPHOS genes down regulated.

A. log2 expression levels of *Cdk6* in pre-B-ALL BCR::ABL1+ patients compared to healthy bone marrow samples. ALL patients n=122, healthy bone marrow n=74. **** p≤0.0001. B. Results of a gene set enrichment analysis of the HALLMARK OXPHOS gene set in *Cdk6$^{KM/KM}$* versus *Cdk6$^{+/+}$* (left panel) and *Cdk6$^{-/-}$* versus *Cdk6$^{+/+}$* (right panel). C. Schematic workflow of CRISPR-Cas9 knock-out of CDK6 in stable BCR::ABL1+ cell lines followed by RNA-seq analysis. D. Normalized enrichment scores of HALLMARK gene sets together with a heatmap of the mean normalized gene expression of genes of the OXPHOS gene set in cells with and without knock-out of CDK6; n= 3 single-cell clones per genotype.

Figure S2. CDK6 binds to promoters of OXPHOS genes.

A. Genomic location of CDK6 ChIP-seq peaks that lie within open chromatin regions. B. Overlap between CDK6 ChIP-seq peaks that lie within open chromatin regions and NRF-1 ChIP-seq peaks. C. Western blot depicting co-immunoprecipitation of NRF-1 (upper panel) and CDK6 (lower panel) in BCR::ABL1+ cell lines. p16^{INK4A} was used as a positive control for the CDK6 co-immunoprecipitation. IN=input, SN=supernatant, IP = immunoprecipitation.

Figure S3. ***Cdk6**$^{KM/KM}$* **cells show mitochondrial dysfunction due to dyscoupled respiration.**

A. Evaluation of the specific oxygen fluxes corrected by the Rox residual respiration. Coupling states: ROUTINE (R), LEAK respiration (L) and ET capacity (E). Values are represented as median ±IQR. n= 6. * p≤0.05, *** p≤0.001.

Figure S4. ***Cdk6**$^{KM/KM}$* **cells have smaller and condensed mitochondria.**

A. Fold change of proliferation of BCR::ABL1+ CRISPR-Cas9 CDK4 knock-out cells to wildtype cell lines after 4 and 7 days of seeding a defined cell number. n= 3 biological replicates per genotype. Data are depicted as mean ± SD; * p≤0.05. B. Mean fluorescence intensity (MFI) of mitotracker stainings of BCR::ABL1+ CRISPR-Cas9 CDK4 knock-out cell lines compared to wildtype lines. n= 3 biological replicates per genotype. Data are depicted as mean ± SD.

Figure S5. ***Cdk6**$^{KM/KM}$* **cells have an increased need for aerobic glycolysis.**

A. ATP levels determined by CellTiter-Glo® Luminescent Cell Viability Assay after separation of mitochondria and cytoplasmatic fractions normalized to the total protein content of the respective fraction; n= 3 cell lines per genotype. Data are depicted as mean ± SD; * p≤0.05. B. Schematic depiction of the Warburg effect where cancer cells switch their metabolism to aerobic glycolysis. They rely on the import of large amounts of glucose and mainly use glycolysis for their energy production. This leads to the accumulation of lactate as an end-product. To prevent intracellular acidification, the high amounts of lactate need to be exported out of the cell. C. Extracellular lactate levels of BCR::ABL1+ *Cdk6*$^{+/+}$ cells treated with palbociclib for two weeks compared to untreated control measured with Lactate-Glo™ Assay; n= 3 biological replicates per condition (rep1-3). D. qPCR analysis of Slc2a3 mRNA levels in BCR::ABL1+ *Cdk6*$^{KM/KM}$ and *Cdk6*$^{-/-}$ cells relative to *Cdk6*$^{+/+}$; n=3 cell lines per genotype. Data are depicted as mean ± SD; * p≤0.05. E. Proliferation experiment of BCR::ABL1+ *Cdk6*$^{+/+}$ cell lines treated with the indicated agents. Cell numbers were determined over time; n= 3 cell lines

per genotype. Data are depicted as mean ± SD. F. Percentage of BCR::ABL1+ cells in G1 phase of the cell cycle (left) or in all stages of the cell cycle (right) on day 13 of the proliferation curve from Fig. S5E determined by propidium iodide staining; n= 3 cell lines per genotype. Data are depicted as mean ± SD; * $p \leq 0.05$, *** $p \leq 0.001$, **** $p \leq 0.0001$. G. AnnexinV/7AAD staining of BCR::ABL1+ cells on day 13 of the proliferation experiment from Fig. S5E; n= 3 cell lines per genotype. Data are depicted as mean ± SD. H. Proliferation experiment of human BCR::ABL1+ SUPB15 cells treated with the indicated agents. Cell numbers were determined over time; n= 3 replicates. Data are depicted as mean ± SD. I. AnnexinV/7AAD stainings of SUPB15 cells on day 14 of the proliferation experiment from Fig. S5H. J. Schematic representation of the HPC^{LSK} and BCR::ABL1+ co-culture competition growth experiment. K. Proliferation of HPC^{LSK} compared to cocultured murine BCR::ABL1+ cells over time treated with the indicated compounds. Depicted is the percentage of GFP- and GFP+ cells corresponding to HPC^{LSK} (GFP-) and BCR::ABL1+ (GFP+) cells, respectively. n=3 replicates per condition per cell line. Data are depicted as mean ± SD.

Figure S6. Combined inhibition of CDK6 and glycolysis increases apoptosis in CML.

A, B. Proliferation experiment of human K562 (A) and AR230 (B) CML cells treated with the indicated agents. Cell numbers were determined over time; n= 4 replicates. C, D. AnnexinV/7AAD stainings of human K562 (C) and AR230 (D) cells on day 13 of the proliferation experiment (see Fig. S6A, S6B). E. Proliferation experiment of human AR230R (imatinib resistant) cells treated with the indicated agents. Cell numbers were determined over time; n= 4 replicates. F. AnnexinV/7AAD stainings of human AR230R cells on day 11 of the proliferation experiment (see Fig. S6E).

Supplementary Materials and Methods

Cell Culture

Generation of BCR::ABL1+ cell lines was performed as described previously (1). In brief, bone marrow cells were isolated from C57BL/6 mice carrying wildtype CDK6 (*Cdk6*$^{+/+}$), a CDK6 mutant with inactivated kinase function (*Cdk6*$^{KM/KM}$) or a CDK6 knock-out (*Cdk6*$^{-/-}$). These cells were transduced with a pMSCV-BCR–ABLp185-IRES-GFP vector. Outgrowth of cell lines was monitored. For re-expression of HA-CDK6 and HA-CDK6K43M in *Cdk6*$^{-/-}$ cell lines, pMSCV-IRES-GFP plasmids were used. CRISPR-knockouts were generated as described previously (2). Briefly, BCR::ABL1+ *Cdk6*$^{+/+}$ cell lines were electroporated using the Neon Transfection System (Invitrogen, Carlsbad, CA, USA) with 1µg pSpCas9(BB)-2A-GFP(px458)-hCDK6del, the plasmid for expression of sgRNAs against CDK6. For each transfection, 20 000 cells were resuspended in Buffer R (Invitrogen) in a reaction volume of 10µl. Transfected cells were cultured for 24h before GFP+ cells expressing the sgRNA construct were single-cell sorted using a FACSAria II cell sorter (BD Biosciences, San Jose, CA, USA). The CDK6 knock-outs were verified on Western Blot. The same procedure was applied to generate CDK4 CRISPR cell lines. All murine BCR::ABL1+ cell lines were maintained in RPMI medium supplemented with 10% FCS, 50 µmol/L 2- mercaptoethanol, 100 U/mL penicillin, and 100 µg/mL streptomycin. Human BCR::ABL1+ SUPB15, K562 and AR230 cell lines were maintained in RPMI medium supplemented with 10% FCS, 100 U/mL penicillin, and 100 µg/mL streptomycin. Imatinib resistant AR230R cells were a kind gift from Michael Deininger (University of Utah, USA) and were retained under the same conditions but media was supplemented with 1µM imatinib. HPC^{LSK} cell lines generated from C57BL/6N mice were established, cultured, and maintained as previously reported (3). Cells were cultivated at 37°C in a 5% CO2 humidified incubator and routinely tested for mycoplasma contaminations.

Analysis of patient data with BCR::ABL1+ ALL

Publicly available patient data from the Microarray Innovations in Leukemia (MILE) study (GSE13159) were analyzed. For this manuscript, we performed differential gene expression analysis using the R package Limma (version 3.54.0) with preprocessing and data normalisation using RMA and the lmFit and eBayes functions.

Microarray gene expression analysis

We revisited previously published gene expression data from mouse bone marrow cells harvested 10 days after retroviral transduction with the *BCR::ABL1* oncogene. Detailed methods on the generation of these data were provided with the previous publication (1) and the dataset was deposited in the Gene Expression Omnibus (GEO) database (accession: GSE87420). In brief, bone marrow of 6 weeks old C57BL/6J mice was isolated and cells were retrovirally transduced with the *BCR::ABL1* oncogene. After 24 hours, 5 x 10^5cells/ml cells were embedded into growth factor-free methylcellulose (MethoCult™, STEMCELL Technologies, Vancouver, Canada). After 10 days, colonies were picked for RNA extraction. Total RNA was isolated from individual colonies using the RNeasy Micro Kit (Qiagen, Hilden, Germany). The quality of the RNA was evaluated using the Laboratory-Chip technique (Agilent Bioanalyzer, Agilent Technologies, Santa Clara, CA, USA). Samples were hybridized to Agilent-074809 SurePrint G3 Mouse GE v2 8x60K microarrays. For this manuscript we performed differential gene expression analysis using the R package Limma (version 3.54.0) with background correction method “normexp”, normalisation method “loess” and the lmFit and eBayes function with default parameters to receive lists of differentially expressed genes comparing $Cdk6^{KM/KM}$ and $Cdk6^{+/+}$ as well as $Cdk6^{-/-}$ and $Cdk6^{+/+}$ colonies.

RNA sequencing of BCR::ABL1+ $Cdk6^{+/+}$ and $Cdk6^{-/-}$ cell lines generated using CRISPR

Samples from BCR::ABL1+ $Cdk6^{+/+}$ cells expressing sgRNAs against CDK6 were processed

for sequencing using the TruSeq RNA Sample Preparation Kit (Illumina Inc., San Diego, CA, USA) as described previously (2). The RNA-seq data reported in this article has been deposited in the GEO database (accession: GSE145220).

RNA sequencing and data analysis of BCR::ABL1+ *Cdk6*$^{+/+}$, *Cdk6*$^{KM/KM}$ and *Cdk6*$^{-/-}$ cell lines generated from mouse bone marrow

Preparation of samples from *Cdk6*$^{+/+}$, *Cdk6*$^{-/-}$ and *Cdk6*$^{KM/KM}$ BCR::ABL1+ cell lines was performed as described previously (1). Libraries for RNA sequencing were generated using the TruSeq RNA Sample Preparation Kit (Illumina Inc.) and were sequenced on an Illumina HiSeq2000 instrument. Reads coming from the same sample sequenced on multiple lanes were merged into a single fastq file. After quality control and adaptor trimming using FASTQC (version 0.11.5) and trimmomatic (version 0.36) reads were aligned against the mm10 mouse reference genome (Gencode version M13) using STAR (version 2.7.6a). Alignments were quality controlled using Qualimap (version 2.2.1). Reads overlapping exons of the mouse Gencode version M13 gene model were then counted using the FeatureCounts function from the Subread package and a count table was generated containing counts per gene and sample. Next, the R package DESeq2 (version 1.30.0) was used to perform differential gene expression analysis between *Cdk6*$^{KM/KM}$ and *Cdk6*$^{+/+}$ samples as well as *Cdk6*$^{-/-}$ and *Cdk6*$^{+/+}$ samples. For each comparison, genes with less than 10 counts across all samples were excluded, then the analysis was performed using the "DESeq" function with default parameters and the *Cdk6*$^{+/+}$ the sample as the reference. The dataset was deposited in the GEO database (accession: GSE156966, GSE266394).

Gene Set Enrichment Analysis (GSEA)

Results from microarray and RNA-seq analyses were subjected to GSEA. Gene ranking was performed by calculating the -log10(p)*sign(log2FC). The gene set database

"h.all.v2023.2.Hs.symbols.gmt" as well as the corresponding dereplicated "Mouse_Ensembl_Gene_ID_Human_Orthologs _MSigDB.v2023.2.Hs.chip" file were used. GSEA was run with the following settings: "remap_Only", "classic" enrichment statistic, "meandiv" normalization and 1000 permutations. Normalized enrichment scores (NES) were plotted using ggplot in R.

ATAC Sequencing

Samples were prepared as previously described (2). *Cdk6*$^{+/+}$, *Cdk6*$^{-/-}$ and *Cdk6*$^{KM/KM}$ BCR-ABL+ cells were resuspended in transposase reaction mix (12.5 µl 2 × TD buffer, 2 µl transposase (Illumina Inc.), 10.5 µl nuclease-free water and 0.01% NP-40). Tagmentation was performed for 30 min at 37 °C. DNA concentration was measured using a Qubit fluorometer (Invitrogen). Libraries were sequenced by the Biomedical Sequencing Facility at the Center for Molecular Medicine (CeMM, Vienna, Austria) using the Illumina HiSeq3000/4000 platform. ATAC-seq data are deposited in the GEO database (accession: GSE156966, GSE266393). ATAC sequencing data were analyzed using the ChIP seq data analysis pipeline described in the Materials and Methods section of the main text with slight alterations as follows: Parameters for peak calling with MACS2 were changed to -- nomodel --shift -100 --extsize 200. We defined genomic regions of open chromatin associated with *Cdk6*$^{+/+}$ or *Cdk6*$^{KM/KM}$ by merging overlapping peak-regions of biological replicate samples. Peaks that were only found in one of the biological replicates were excluded.

Integrative analysis of ChIP seq, ATAC seq and RNA seq data

ChIP seq-peak regions for the NRF-1 transcription factor were published by the ENCODE project and downloaded from https://www.encodeproject.org/files/ENCFF153XGN/ (last accessed 31.10.2023). Promoters of "OXPHOS genes" were defined as follows: First, human gene symbols in the HALLMARK_OXIDATIVE_PHOSPHORYLATION gene set (MSigDB

v.7.2) were mapped to mouse Ensembl gene IDs using the mapping provided in the Mouse_ENSEMBL_Gene_ID_Human_Orthologs_MSigDB.v7.2.chip file (MSigDB v. 7.2) after removal of genes with duplicate mappings. For the resulting set of 177 mouse genes, all transcripts included in the GENCODE vM13 primary assembly gene model were extracted. Promoters were defined as the genomic regions from 2000 bp upstream to 200 bp downstream of the transcriptional start site of each of the transcripts. CDK6 ChIP seq-peak regions overlapping open chromatin regions, NRF-1 binding sites or OXPHOS gene promoters were identified using the findOverlaps function of the R package GenomicRanges (version 1.42.0).

Transcription factor motif analysis

The HOMER software package (version 4.9.0) was used to investigate transcription factor motif enrichment. Genomic regions of interest were analyzed for *de novo* motif enrichment against the mouse Gencode M13 primary assembly sequence with the findMotifsGenome.pl script using default parameters.

RNA Isolation and RT-qPCR

Total RNA from cell lines was isolated using the RNeasy Mini Kit (Qiagen) according to manufacturer's instructions. For reverse transcription, the iScript cDNA Synthesis Kit (Bio-Rad, Hercules, CA, USA) was used. Real-time quantitative PCRs (RT-qPCR) were performed with SsoAdvanced Universal SYBR Green Supermix (Bio-Rad) on a CFX96 real time PCR cycler (Bio-Rad). Expression levels were normalized to Rplp0 mRNA levels. Data were analyzed with Graphpad Prism. Ordinary one-way ANOVA test with multiple comparisons was performed to evaluate statistical significance.

High-resolution respirometry

High-resolution respirometry (HRR) has been performed using Oxygraph-2k-

FluoRespirometer (O2k, Oroboros Instruments, Innsbruck, Austria) devices. This system allows to measure mitochondrial respiration in living cells. Briefly, *Cdk6*$^{+/+}$, *Cdk6*$^{-/-}$ and *Cdk6*$^{KM/KM}$ BCR::ABL1+ cells were trypsinized, suspended in 5 mL fresh medium, and counted in the CASY® Model TT cell counter (Schärfe-System, Reutlingen, Germany). After, the cells were placed in the O2k chamber in a concentration of 1x10^6 cells/mL. O2ks were previously air-calibrated in the day of the experiment. Machine settings were adjusted to 37°C and stirring of 750 rpm. For calibration with cell culture medium the O_2 solubility factor was set at 0.89 (4). O_2 background and zero calibrations were performed monthly to allow reliable oxygen consumption measurements (5). O_2 concentration [µM] and O_2 flux [amol • s^{-1} • x^{-1}] were recorded in real time with the DatLab 7.4 software (Oroboros Instruments). The protocol used for evaluation of the coupling-control states was the SUIT-003_O2_ce_D009 for living cells (6). This protocol allows to analyze the coupling control states: ROUTINE (*R*), LEAK (*L*) and electron-transfer pathway capacity (*E*). Cell addition into the O2k chambers was performed by complete volume replacement of the sample. *R* respiration is the physiological coupling state controlled by ATP demand and coupling efficiency. After addition of 10 nM oligomycin (Omy) to get the *L* state. Then, the stepwise titration of the uncoupler Carbonyl cyanide m- chlorophenyl hydrazone, CCCP (U) allowed to get the *E* state. Finally, 0.5 µM rotenone (Rot) and 2.5 µM antimycin A (Ama) were added to inhibit the ET pathway and get the residual O_2 consumption (*Rox*). Respiratory rates were corrected for the instrumental O_2 background flux, dilution of the sample by titrations, and *Rox*. Data evaluation was performed using DatLab 7.4. The p-values for assessing statistical significance levels were determined using the non- parametric unpaired Mann-Whitney t-test. To account for multiple comparisons, a two-way ANOVA with Bonferroni's multiple comparison test was employed specifically for respiratory control coupling states. GraphPad Prism 6 software was utilized to conduct the statistical analyses.

Metabolite analysis

Frozen pellets of *Cdk6*$^{+/+}$, *Cdk6*$^{-/-}$ and *Cdk6*$^{KM/KM}$ BCR::ABL1+ cells were thawed on ice and extracted using ice-cold 80% methanol, and cleared extracts were dried under nitrogen. Samples were taken up in MS-grade water and mixed with the heavy isotope labelled internal standard mix. A 1290 Infinity II UHPLC system (Agilent Technologies) coupled with a 6470 triple quadrupole mass spectrometer (Agilent Technologies) was used for the LC-MS/MS analysis. The chromatographic separation for samples was carried out on a ZORBAX RRHD Extend-C18, 2.1 x 150 mm, 1.8 um analytical column (Agilent Technologies). The column was maintained at a temperature of 40°C and 4 µL of sample was injected per run. The mobile phase A was 3% methanol (v/v), 10 mM tributylamine, 15 mM acetic acid in water and mobile phase B was 10 mM tributylamine, 15 mM acetic acid in methanol. The gradient elution with a flow rate of 0.25 mL/min was performed for a total time of 24 min. Afterwards back-flushing of the column using a 6-port/2-position divert valve was carried out for 8 min using acetonitrile, followed by 8 min of column equilibration with 100% mobile phase A. The triple quadrupole mass spectrometer was operated in negative electrospray ionization mode, spray voltage 2 kV, gas temperature 150 °C, gas flow 1.3 L/min, nebulizer 45 psi, sheath gas temperature 325 °C, sheath gas flow 12 L/min. The metabolites of interest were detected using a dynamic MRM mode. MassHunter 10.0 software (Agilent Technologies) was used for data processing. Ten-point calibration curves with internal standardization were constructed for the quantification of metabolites. One-way or two-way ANOVA with Bonferroni multiple-comparison test analysis were performed to evaluate statistical significance.

Mitotracker

40 000 cells were stained with 20nM MitoTracker Deep Red FM (Invitrogen) and incubated 30 minutes at 37°C. Mitochondrial mass was determined on CytoFLEX S (Beckman Coulter, Fullerton, CA, USA). Data were analyzed using the CytExpert software and Graphpad Prism.

Paired, two-tailed t-test was performed to evaluate statistical significance between *Cdk4*$^{+/+}$ and *Cdk4*$^{-/-}$. Ordinary one-way ANOVA test with multiple comparisons was performed to evaluate statistical significance between *Cdk6*$^{+/+}$, *Cdk6*$^{-/-}$ and *Cdk6*$^{KM/KM}$.

ATP levels

15 000 cells were seeded two hours before measuring total ATP levels with CellTiter-Glo assay (Promega Corporation, Fitchburg, WI, USA) according to manufacturer's instructions. Mitochondrial and cytoplasmic ATP levels were measured after the separated fractions were diluted 1:100 in PBS. Luminescence was determined on EnSpire plate reader (PerkinElmer, Waltham, MA, USA). Data were analyzed with Graphpad Prism. Statistical significance was analyzed using linear regression in R with genotype as the predictor variable, an additional variable accounting for "batch" was added to the model.

Mitochondria Isolation

Mitochondrial and cytoplasmatic fraction from $2x10^7$ cells were separated using Mitochondria Isolation Kit for Cultured Cells (Thermo Scientific, Rockford, IL, USA). Isolation was performed according to manufacturer's instructions using the reagent-based method. Protein levels of the fractions were determined using the Pierce™ BCA Protein Assay (Thermo Scientific, Rockford, IL, USA).

Extracellular lactate levels

Cells were either untreated or treated with 500nM palbociclib for two weeks with refreshment of the treatment every 48 hours. 10 000 cells were seeded in FCS-free media one hour before measurements. 25µl of supernatant were used to determine extracellular lactate levels with LactateGlo Assay (Promega). Equal volumes of LactateGlo Detection Reagent were added and samples were incubated for one hour before measuring luminescence on EnSpire plate reader (Perkin Elmer). Data were analyzed with Graphpad Prism. Statistical significance was

analyzed using linear regression in R with genotype as the predictor variable (Fig. 5D) or via paired t-test (Fig. S5C).

Competitive proliferation assay

2000 HPC^{LSK} and 10 BCR::ABL1-GFP+ cells (200:1 ratio) were co-cultured and single treated either with 250nM palbociclib or 125µM 2-DG or co-treated with both compounds for 10 days. Treatments were refreshed after 48 hours. Cell numbers and ratio of GFP+ and GFP- cells were determined regularly with the CytoFLEX S (Beckman Coulter, Fullerton, CA, USA). Data were analyzed using CytExpert and Graphpad Prism. To analyze statistical significance, the relative fraction of HPC^{LSK} compared to BCR::ABL1+ was modelled as a function of treatment using a linear model in R.

RNA sequencing and data analysis of CML patients

RNA-seq analyses were performed on a CML patient cohort described previously (7). In brief, total RNA was extracted from mononuclear cells using RNeasy kit (Qiagen) and processed for sequencing using the TruSeq RNA Sample Preparation Kit (Illumina Inc) as recommended by the manufacturers. Raw sequencing reads were quality processed using Trim_galore followed by alignment to GRCh38 using STAR (8). Differential expression between groups was calculated using the DESeq2 algorithm. VST transformed expression values were used for data visualization. RNA-seq data were deposited at GEO database (accession: GSE267912).

Co-Immunoprecipitation

10x10^6 *Cdk6*$^{-/-}$, *Cdk6*$^{KM/KM}$ and *Cdk6*$^{+/+}$ or HA-Cdk6 (a-NRF-1 IP) cells per sample were lysed in ELB buffer (0.1% NP-40, 50 mM N-2-hydroxyethylpiperazine-N'-2-ethanesulfonic acid (HEPES), 250 mM NaCl, 5mM EDTA and proteinase inhibitors (cOmplete protease inhibitor

cocktail, Roche Diagnostics, Indianapolis, IN, USA)) by snap-freezing in liquid nitrogen. Lysates were thawed on ice and pre-cleared with agarose beads (Pierce Protein A/G Agarose beads; Thermo Scientific) at 4°C for 2 hours under rotation. Protein concentration was determined via Bradford assay. 300 µg protein lysates were used for co-immunoprecipitation. 8µg antibody (CDK6 PA5-27978, or NRF-1 PA5-27854, Invitrogen) were added to each sample before incubation under rotation at 4°C overnight. Then, 40 µl agarose beads per sample were pre-washed with ELB buffer and added to the samples. Samples were incubated for 2 hours at 4°C under rotation. Beads were pelleted and separated from supernatant. Supernatant was saved as control. Beads were washed in ELB buffer before elution with 4x Laemmli buffer (100 mM Tris/HCl pH 6.8, 2.5% sodium dodecyl sulfate (SDS), 10% glycerol) for 10 minutes at 95°C. Eluates were analyzed by SDS-PAGE and immunoblot.

Immunoblotting

Protein lysates in Laemmli buffer were separated by SDS/PAGE and transferred onto nitrocellulose blotting membrane (Amersham™ Protran™ 0.45µm NC, GE Healthcare, Life Sciences) using the TransBlot Turbo Transfer System (Bio-Rad). Membranes were blocked in 5% milk followed by incubation with primary antibodies: anti-CDK6 (1:1000, H-96 sc-7180, Santa Cruz Biotechnology, Dallas, TX, USA) and anti-NRF-1 (1:500, G-5 sc-515360, Santa Cruz Biotechnology), anti-p16^{INK4a} (1:1000, ab211542, abcam, Cambridge, UK). Secondary HRP-coupled antibodies (1:10000, Cell Signaling Technology, Danvers, MA, USA) were used and after addition of Clarity™ ECL Western blotting substrate (Bio-Rad), chemiluminescence was detected with ChemiDoc Touch Imaging System (Bio-Rad). Original uncropped Western Blot images can be found in the Supplementary data.

Supplementary References

1. Bellutti F, Tigan A-S, Nebenfuehr S, Dolezal M, Zojer M, Grausenburger R, et al. CDK6 Antagonizes p53-Induced Responses during Tumorigenesis. Cancer Discov. 2018 Jul;8(7):884–97.

2. Heller G, Nebenfuehr S, Bellutti F, Ünal H, Zojer M, Scheiblecker L, et al. The Effect of CDK6 Expression on DNA Methylation and DNMT3B Regulation. iScience. 2020 Oct 10;23(10):101602.

3. Doma E, Mayer IM, Brandstoetter T, Maurer B, Grausenburger R, Menzl I, et al. A robust approach for the generation of functional hematopoietic progenitor cell lines to model leukemic transformation. Blood Adv. 2021 Jan 12;5(1):39–53.

4. Gnaiger E. Polarographic oxygen sensors, the oxygraph and high-resolution respirometry to assess mitochondrial function. Drug-Induced Mitochondrial Dysfunction. John Wiley Sons, Inc. 2008;327–52.

5. Gnaiger E. Mitochondrial Pathways and Respiratory Control An Introduction to OXPHOS Analysis. Bioenerg Commun. 2020;2.

6. Doerrier C, Garcia-Souza LF, Krumschnabel G, Wohlfarter Y, Mészáros AT, Gnaiger E. High-resolution fluorespirometry and oxphos protocols for human cells, permeabilized fibers from small biopsies of muscle, and isolated mitochondria. Methods Mol Biol. 2018;1782:31–70.

7. Heller G, Topakian T, Altenberger C, Cerny-Reiterer S, Herndlhofer S, Ziegler B, et al. Next-generation sequencing identifies major DNA methylation changes during progression of Ph+ chronic myeloid leukemia. Leukemia. 2016 Sep 1;30(9):1861–8.

8. Dobin A, Davis CA, Schlesinger F, Drenkow J, Zaleski C, Jha S, et al. Sequence analysis STAR: ultrafast universal RNA-seq aligner. Bioinformatics. 2013;29(1):15–21.

Supp. Figure 1

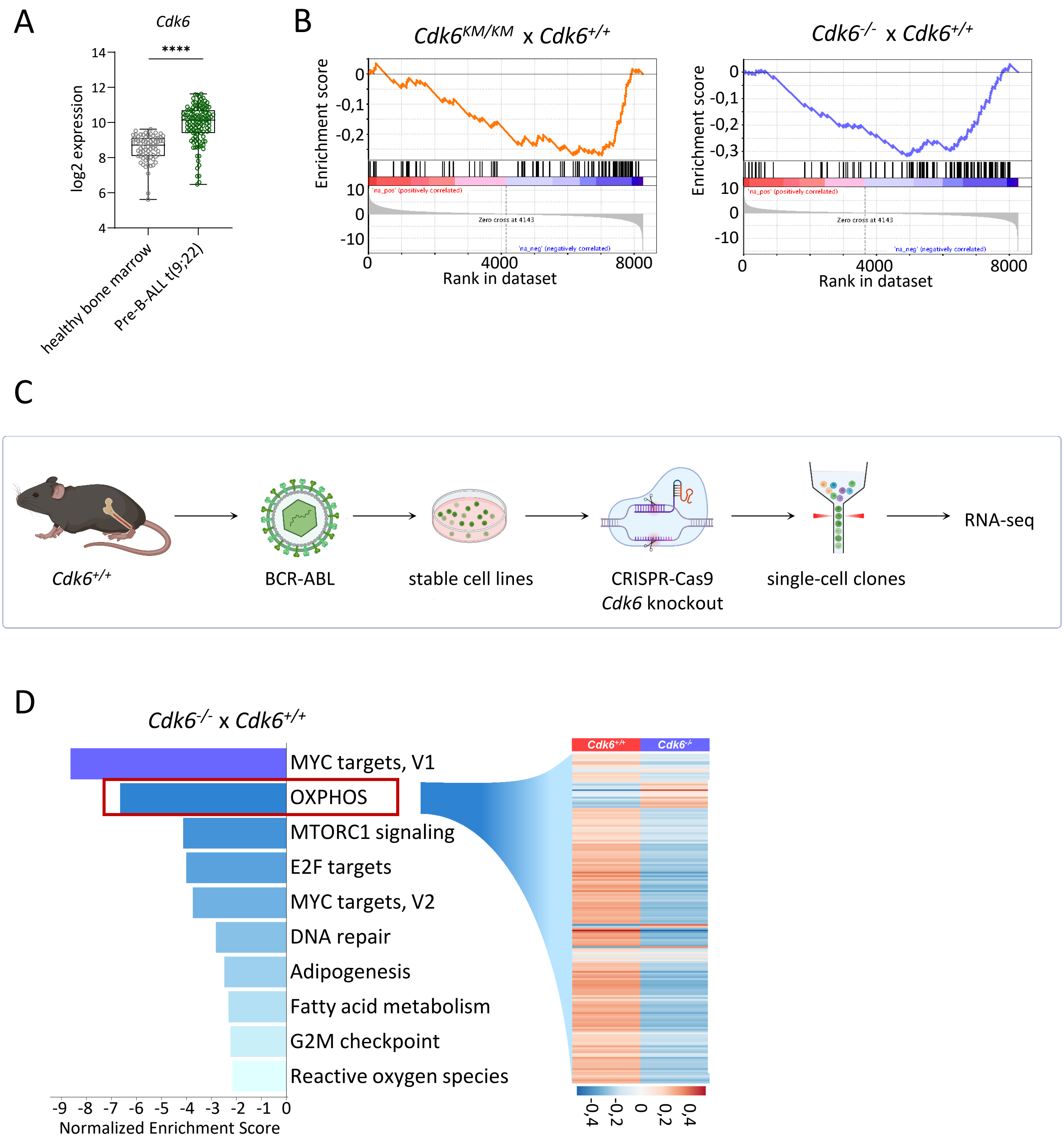

Supp. Figure 2

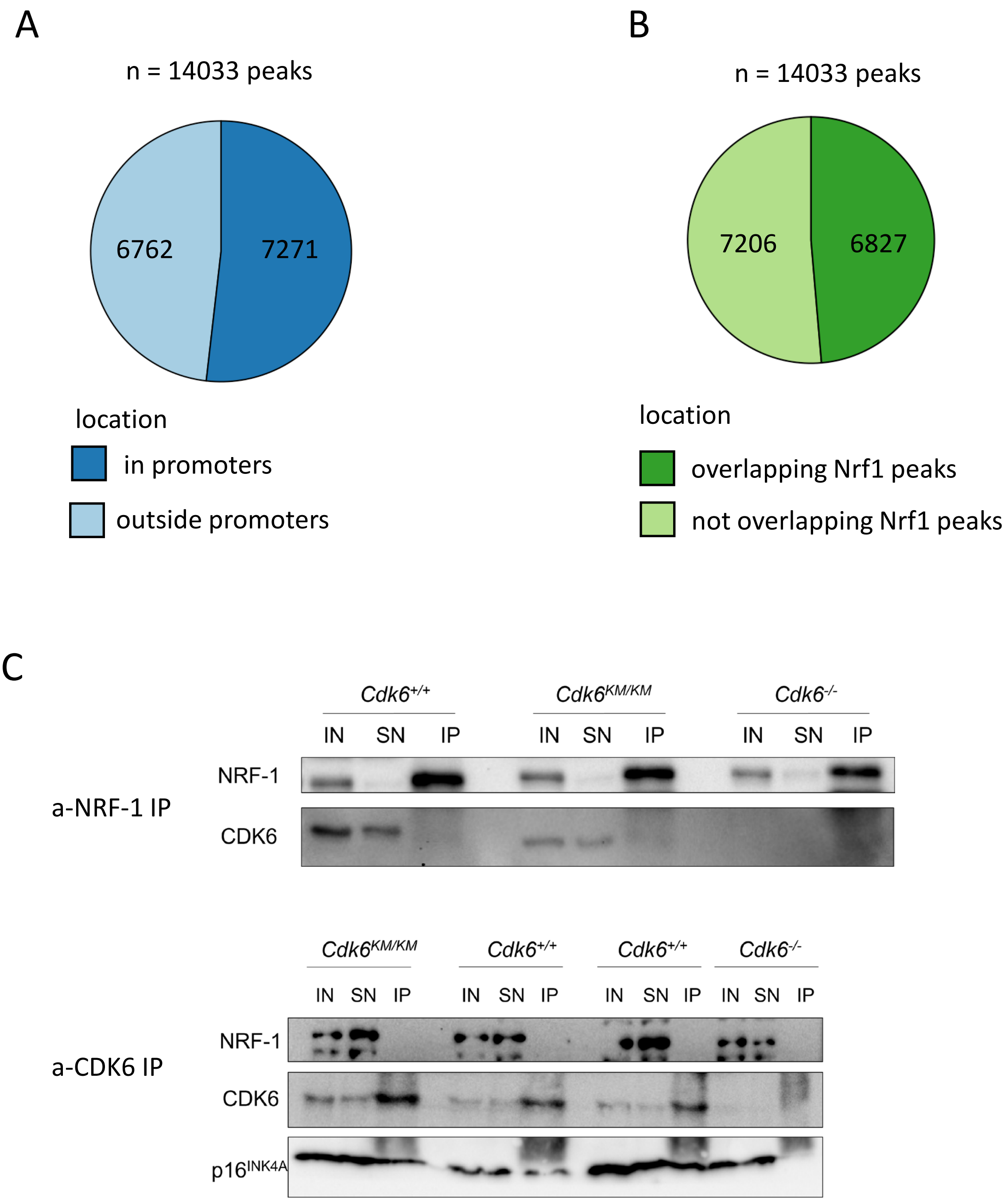

Supp. Figure 3

A

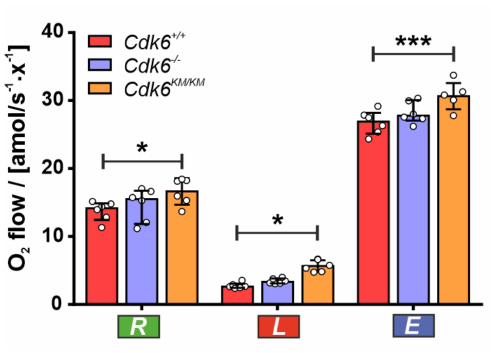

Supp. Figure 4

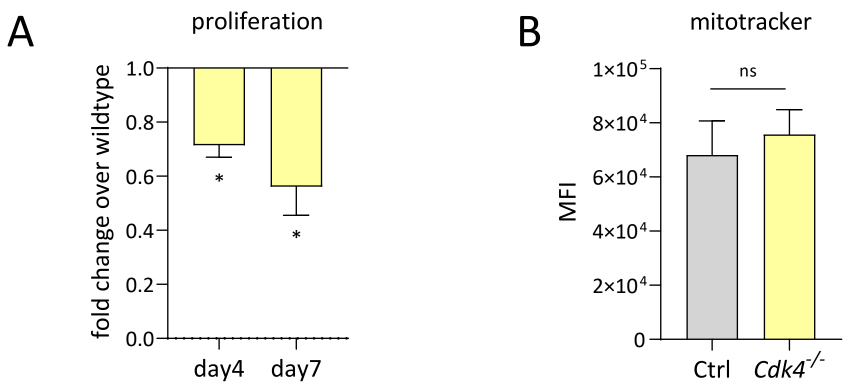

Supp. Figure 5

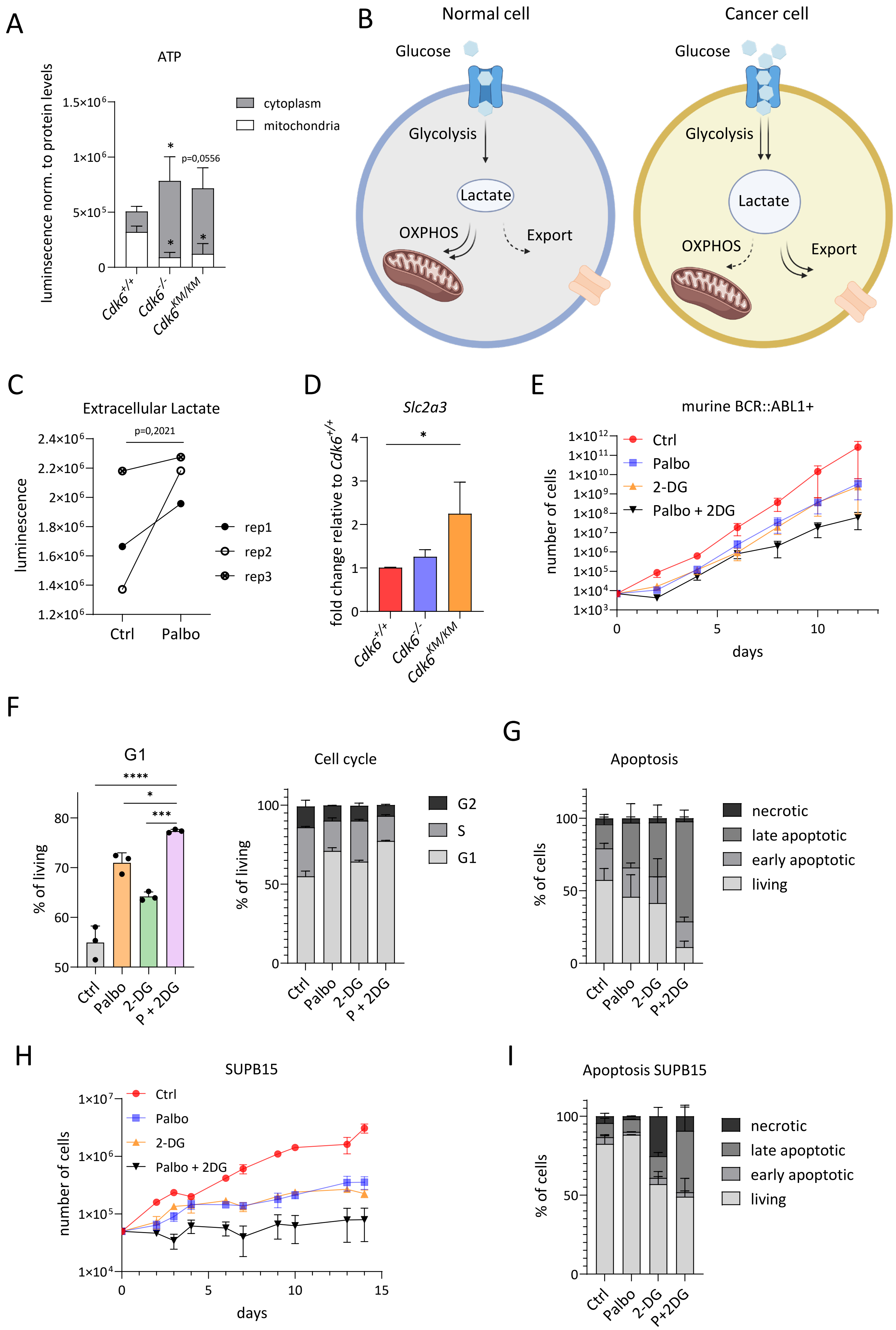

Supp. Figure 5

J

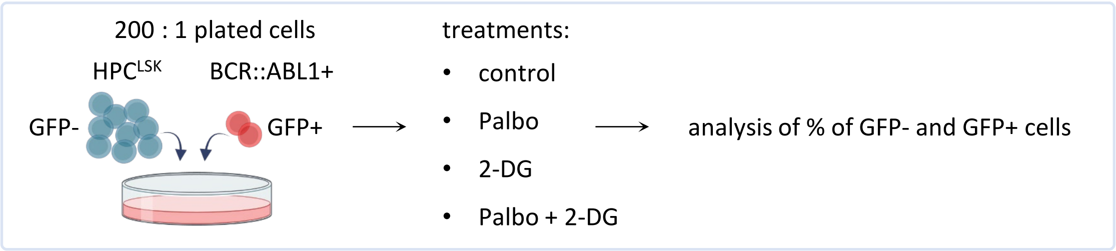

K

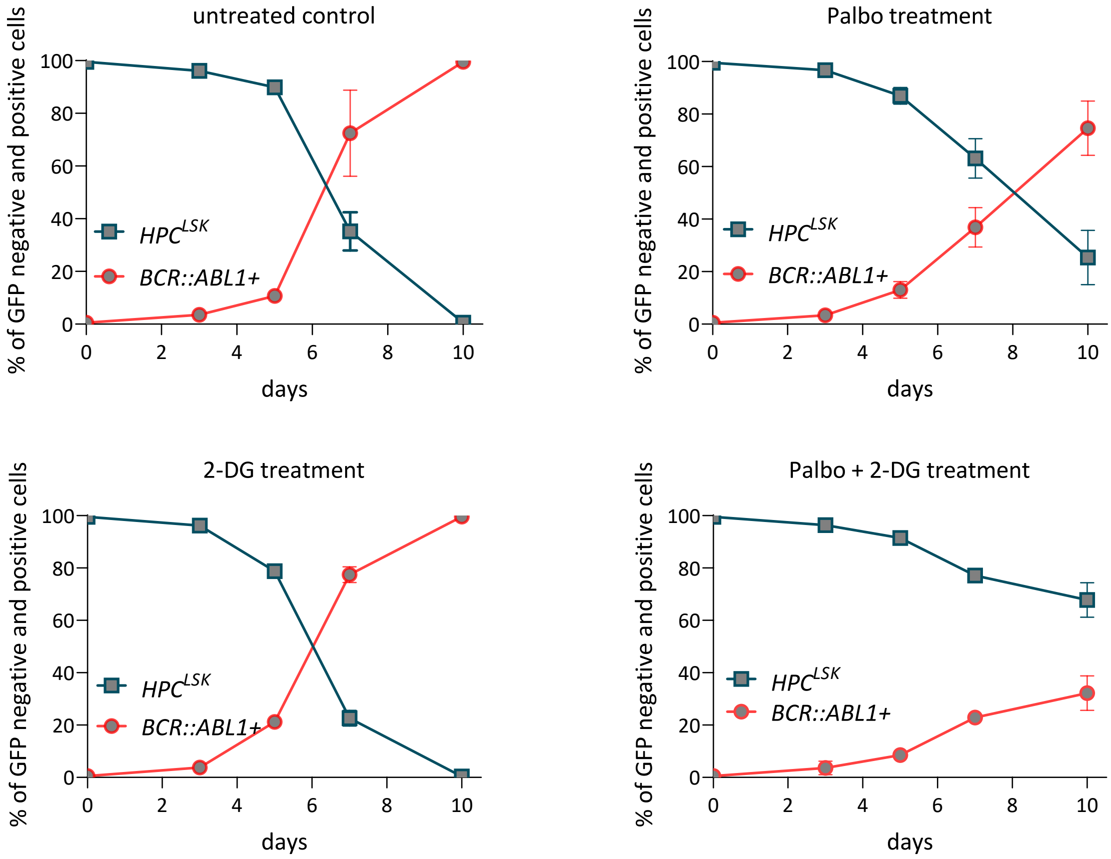

Supp. Figure 6

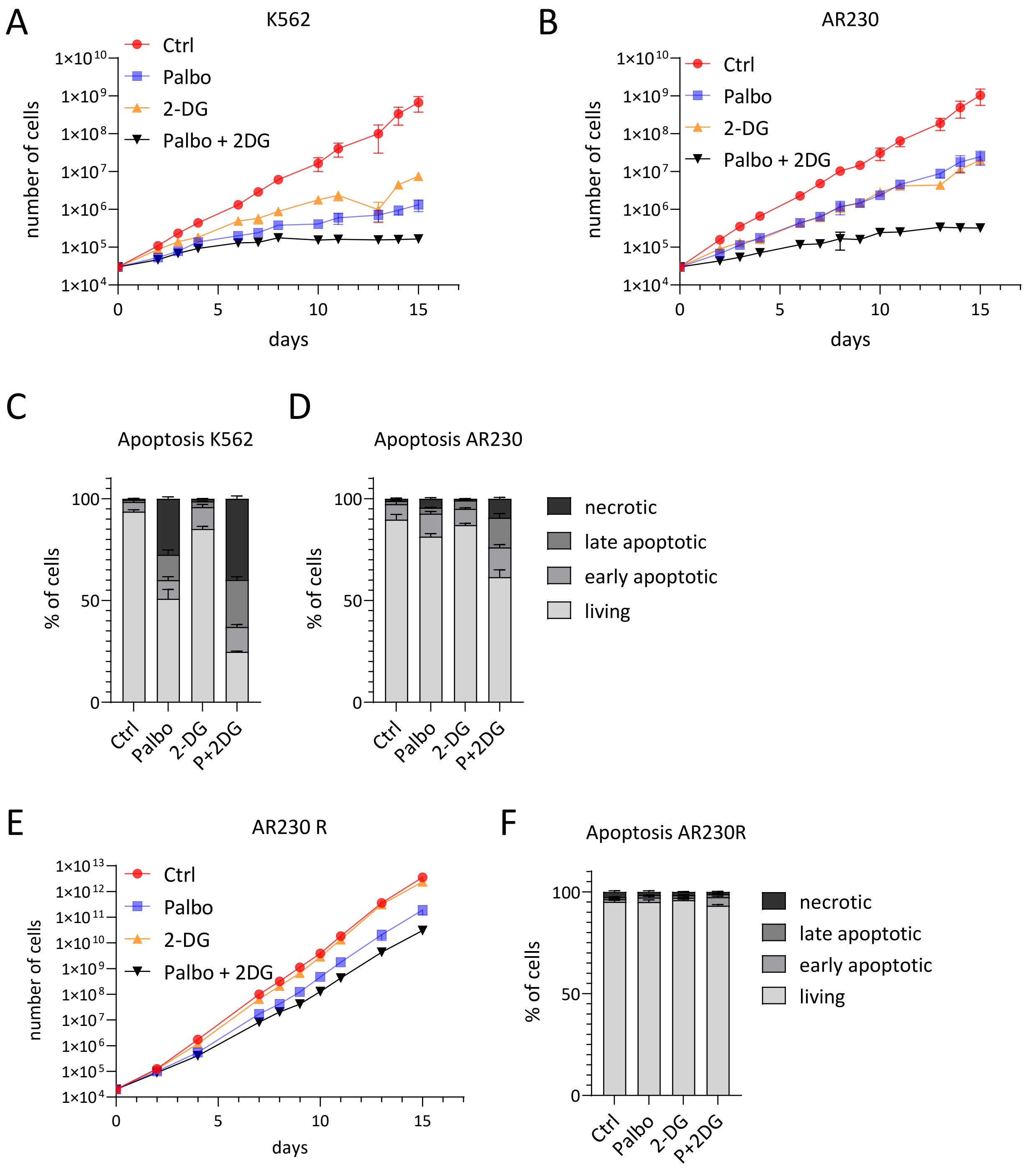

original data:

Immunoblot CDK6 corresponding to Supp Fig 2C, lower panel

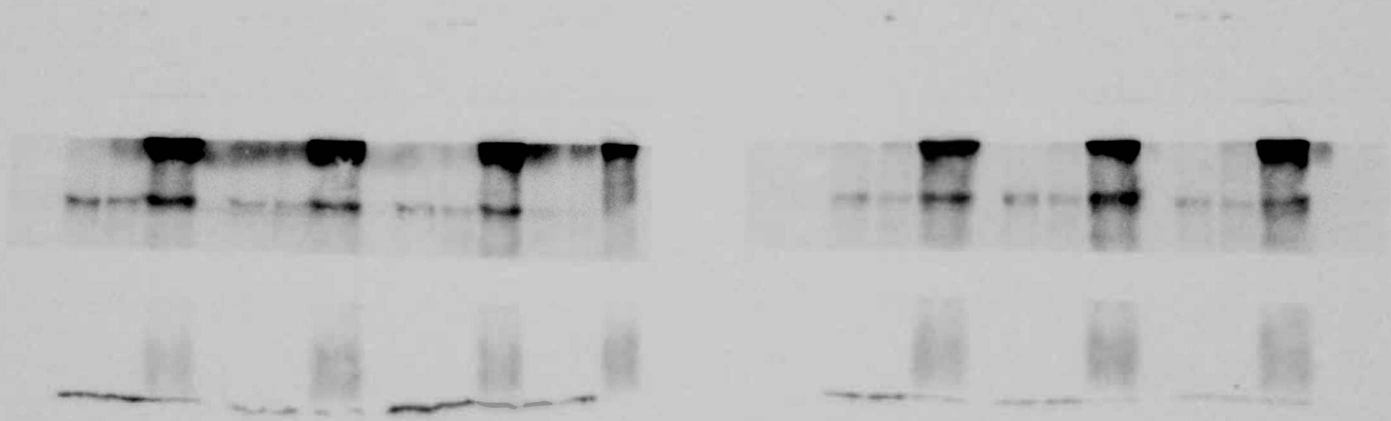

Immunoblot NRF1 corresponding to Supp Fig 2C, lower panel

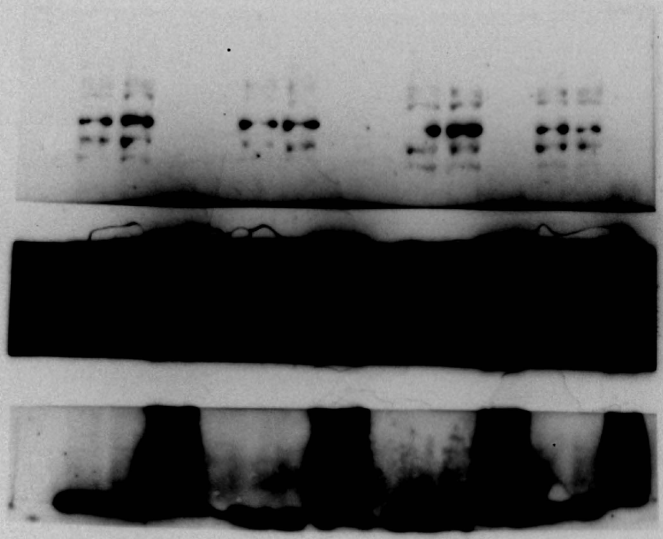

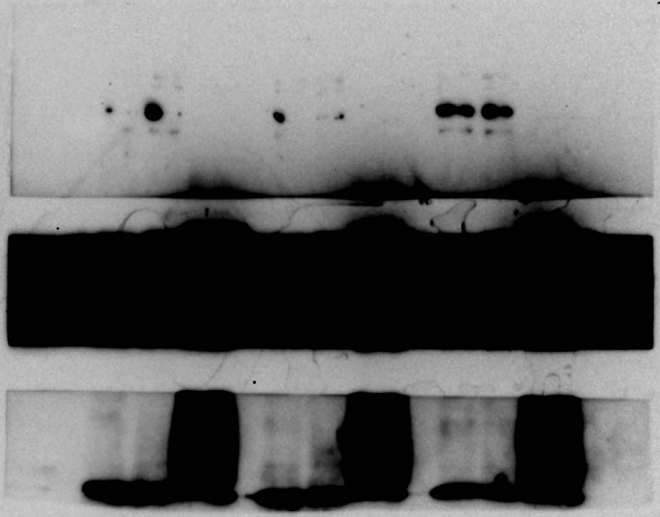

Immunoblot p16 corresponding to Supp Fig 2C, lower panel

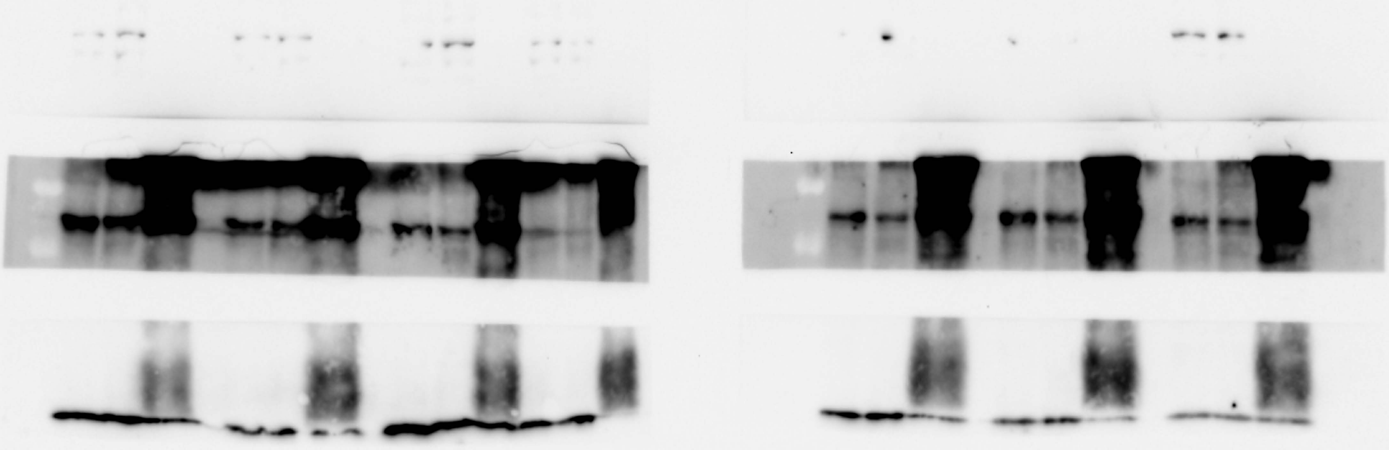

Immunoblot CDK6 corresponding to Supp Fig 2C, upper panel

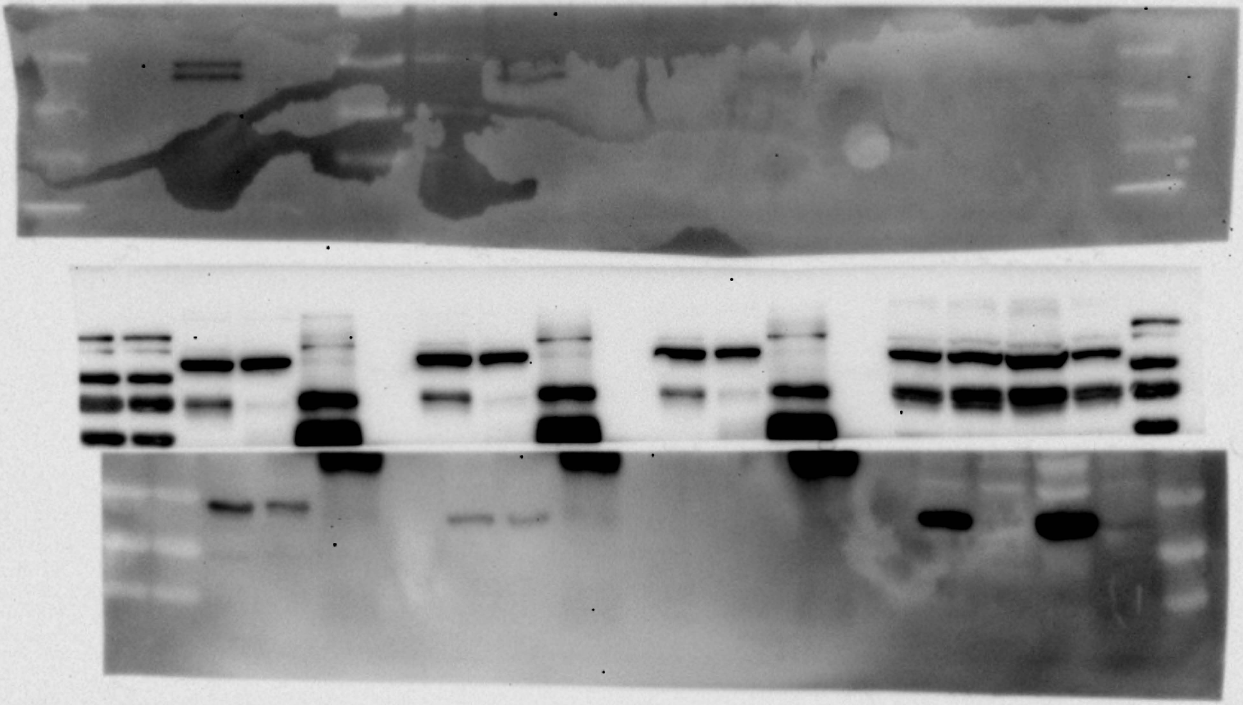

Immunoblot NRF1 corresponding to Supp Fig 2C, upper panel

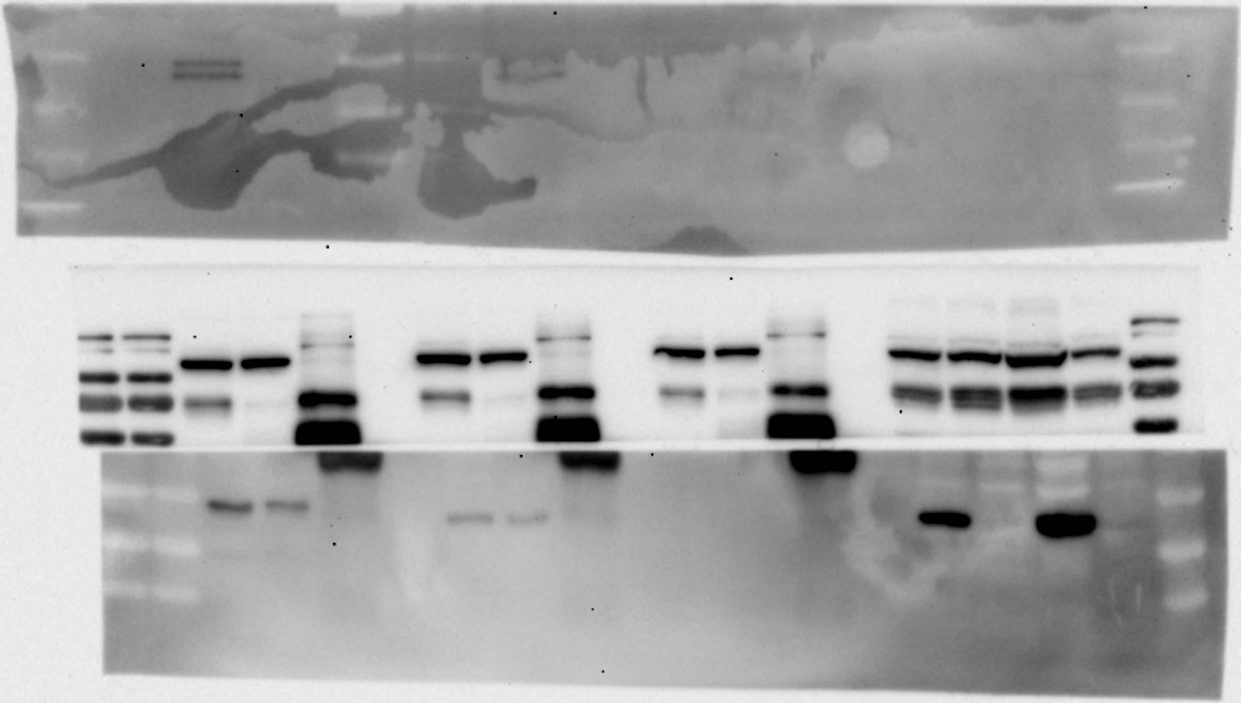

Supplement: Supplementary file 1 — supplemental material [file 41419_2025_7434_MOESM1_ESM.pdf]
